# A Cell‐Free Platform Based on Nisin Biosynthesis for Discovering Novel Lanthipeptides and Guiding their Overproduction In Vivo

**DOI:** 10.1002/advs.202001616

**Published:** 2020-07-21

**Authors:** Ran Liu, Yuchen Zhang, Guoqing Zhai, Shuai Fu, Yao Xia, Ben Hu, Xuan Cai, Yan Zhang, Yan Li, Zixin Deng, Tiangang Liu

**Affiliations:** ^1^ Key Laboratory of Combinatorial Biosynthesis and Drug Discovery Ministry of Education and Wuhan University School of Pharmaceutical Sciences Wuhan 430071 China; ^2^ Department of Clinical Laboratory Renmin Hospital of Wuhan University Wuhan 430060 China; ^3^ Hubei Engineering Laboratory for Synthetic Microbiology Wuhan Institute of Biotechnology Wuhan 430075 China

**Keywords:** cell‐free protein synthesis, genome mining, lanthipeptides, lanthipeptides overproduction, lanthipeptides screening

## Abstract

Lanthipeptides have extensive therapeutic and industrial applications. However, because many are bactericidal, traditional in vivo platforms are limited in their capacity to discover and mass produce novel lanthipeptides as bacterial organisms are often critical components in these systems. Herein, the development of a cell‐free protein synthesis (CFPS) platform that enables rapid genome mining, screening, and guided overproduction of lanthipeptides in vivo is described. For proof‐of‐concept studies, a type I lanthipeptide, nisin, is selected. Four novel lanthipeptides with antibacterial activity are identified among all nisin analogs in the National Center for Biotechnology Information (NCBI) database in a single day. Further, the CFPS platform is coupled with a screening assay for anti‐gram‐negative bacteria growth, resulting in the identification of a potent nisin mutant, M5. The titers of nisin and the nisin analog are found to be improved with CFPS platform guidance. Owing to the similarities in biosynthesis, the CFPS platform is broadly applicable to other lanthipeptides, thereby providing a universal method for lanthipeptide discovery and overproduction.

## Graphical abstract

The development of a cell‐free protein synthesis (CFPS) platform is described for rapid genome mining, screening, and the guiding overproduction of lanthipeptides in vivo. The functionality of the CFPS platform is demonstrated by using nisin (a typical lanthipeptide) for proof‐of‐concept studies. This CFPS platform provides a universal method for lanthipeptide discovery and overproduction.

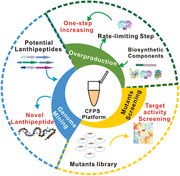

## 1 Introduction

Lanthipeptides are a major group of ribosomally synthesized and posttranslationally modified peptides (RiPPs) produced by microorganisms, characterized by intramolecular thioether crosslinks (termed lanthionine and methyllanthionine residues), with many defined biological activities.^[^ ^1^ ^]^ Genes related to the biosynthesis of lanthipeptides are typically assembled in gene clusters, which encode a precursor peptide (LanA) (comprising a C‐terminal core peptide and a N‐terminal leader peptide), posttranslational modification (PTM) enzymes, transporters, processing proteases, immunity proteins, and regulatory machinery.^[^ ^2^ ^]^ During their biosynthesis, the lanthionine and methyllanthionine residues are introduced in a two‐step PTM process.^[^ ^1^ ^]^ In the first step, Ser and Thr residues in the core peptide of LanA are dehydrated by dehydrase. The thioether crosslinks are then subsequently formed via a Michael‐type addition by Cys residues onto the dehydroamino acids. The LanA with thioether crosslinks is defined as modified precursor peptide (mLanA) and generally lack biological activities. Furthermore, following protease cleavage the leader peptide in mLanA, the remaining core peptide with thioether crosslinks functions to exert various biological activities and is designated as mature lanthipeptides. Because most lanthipeptides exhibit antimicrobial activity, microbes must express transporters and immunity proteins in their biosynthetic gene cluster to achieve adequate protection.

Lanthipeptides hold much promise for the discovery of novel bioactive compounds. However, although their biosynthetic gene clusters have been identified in the genomes of many microorganisms,^[^ ^3^ ^]^ a large proportion of these microorganisms are difficult to culture in the laboratory. Hence, the heterologous synthesis of inactive mLanA (e.g., the coexpression of LanA and PTM enzymes) in *Escherichia coli* and *Lactococcus lactis* and formation of mature lanthipeptides by protease cleavage in vitro provide an opportunity for lanthipeptides synthesis.^[^ ^4^, ^5^ ^]^ Furthermore, construction of a mutant lanthipeptides library and performing high‐throughput screening is another useful method for discovering novel lanthipeptides. Methods have been developed that allow for the high‐throughput screening of new bioactive (antiviral) lanthipeptides in vivo,^[^ ^6^ ^]^ and for ingenious high‐throughput screening of antimicrobial lanthipeptides obtained by expression of inactive mLanAs in living cells and then removal of the leader peptide in vitro (including in dead cells).^[^ ^7^ ^]^ However, the in vivo systems pose unavoidable challenges: intracellular toxicity; frequent formation of inclusion bodies by LanA or mLanA in vivo, which is problematic for subsequent purification; and lack of special tRNA^Glu^ for heterologous dehydrase (dehydration in the lanthipeptide PTM process is catalyzed in a tRNA^Glu^‐dependent manner). Specifically, Hudson et al. reported that *E. coli* tRNA^Glu^ contributes to the lack of dehydrase activity in thiomuracin (a type of RiPPs was synthesized by *Thermobispora bispora*) heterologous biosynthesis.^[^ ^8^ ^]^

In addition to discovering novel lanthipeptides, there is an urgent need for increased production of these molecules as they have a wide range of applications in industry and medicine.^[^ ^9^ ^]^ It has been reported that screening for optimal strains,^[^ ^10^ ^]^ optimizing culture conditions,^[^ ^11^ ^]^ and metabolic engineering^[^ ^12^ ^]^ would serve to improve the production of lanthipeptides. Although the biosynthesis of lanthipeptides involves similar pathways, including LanA synthesis, PTM catalyzation, proteolysis, and export, the rate‐limiting steps of biosynthesis are unknown and no clear principle has been defined to increase production in vivo.

Because many lanthipeptides are bactericidal, cell‐free protein synthesis (CFPS), which is independent of cell growth,^[^ ^13^ ^]^ is a promising approach for lanthipeptides research. Cheng et al. used a commercial in vitro rapid translation system kit to express the nisin (class I lanthipeptide) precursor peptide gene (*nisA*) and PTM genes (*nisB* and *nisC*) together to form modified NisA (mNisA). Active nisin was then obtained by commercial protease trypsin treatment.^[^ ^14^ ^]^ Additional studies on RiPPs synthesis used cell‐free systems to transcribe and translate precursor peptide genes, combined with some of the original PTM purified enzymes in the original RiPPs biosynthetic gene cluster and some heterologous isozymes (the original is difficult to purify or may result in isolation of inactive enzymes); one of these studies even employed a chemical reagent (H_2_O_2_ to generate dehydroalanies) to synthesize RiPPs in vitro.^[^ ^15^ ^]^ These studies demonstrate the flexibility and robustness of CFPS; however, they were unable to fully reconstitute the biosynthesis of RiPPs in microbes and thus, are not capable of identifying the rate‐limiting steps in vivo for RiPPs overproduction, or developing methods for high‐throughput discovery of novel RiPPs in CFPS.

To further explore the methodological advantages of cell‐free systems in the research of lanthipeptides, we developed a CFPS platform that solves the current issues in genome mining and screening of novel antimicrobial lanthipeptides. Furthermore, we achieved overproduction of lanthipeptides via the CFPS platform guidance. Nisin was selected for our proof‐of‐concept experiments as it is the first commercially available lanthipeptide, classified as type I, that has been used as a food preservative worldwide for more than 70 years without the development of bacterial resistance, and has a well‐defined catalytic mechanism in its biosynthesis.^[^ ^5^, ^16^, ^17^ ^]^ We developed an *Escherichia coli* CFPS platform using a simple preparation method to fully reconstruct the natural nisin biosynthetic pathway, and then optimized the ratio of biosynthetic enzymes for higher efficiency. To test the functionality of this optimized CFPS platform, we first performed genome mining on all potential nisin analogs in the NCBI database using our CFPS platform in a single day. We next developed a screening process for the identification of lanthipeptides that are functionally active against gram‐negative bacteria and applied it to our CFPS platform to assess the nisin mutant library. Thirdly, we employed one‐step metabolic engineering in vivo to overproduce nisin in an industrial host, and nisin analogs in a heterologous expression host; the engineered target was identified using the optimized nisin CFPS platform.

## 2 Results

### 2.1 A Cell‐Free Protein Synthesis Platform for Lanthipeptide Biosynthesis

Inactive mNisA has been successfully synthesized using a commercial *E. coli‐*based CFPS, and the active nisin was obtained by commercial protease trypsin cleavage of the leader peptide in mNisA.^[^ ^14^ ^]^ However, because the natural biosynthetic system of nisin is not completely reconstituted in CFPS (lacks original protease NisP), it cannot be employed to identify the rate‐limiting step of nisin biosynthesis in vivo, nor can be used to enhance the CFPS efficiency for nisin synthesis by systematic titration of nisin's components (precursor peptide NisA, PTM enzyme NisB and NisC, and protease NisP), whereas using commercial kits to perform large‐scale screening is costly. We previously developed an *E. coli*‐based cell‐free system,^[^ ^18^ ^]^ which comprises *E. coli* BL21(DE3) cell extracts (lysate protein content 26.5 mg mL^−1^), essential substrates, salts, and cofactors (e.g., amino acids, zinc, dNTPs, CoA) required for the transcription, translation, and PTM of nisin as previously described in detail, which was proven to lack any remaining living cells (Figure S1, Supporting Information). By comparing this cell‐free system with a commercial cell‐free synthesis kit, the result (Figure S2, Supporting Information) showed our system was acceptable for subsequent research. Therefore, we constructed the following four plasmids to achieve nisin Z biosynthesis in our CFPS system: pJL1‐*nisZ* for expression of precursor peptide NisZ; pET28a‐*nisB* and pET28a‐*nisC* for expression of PTM enzymes NisB and NisC, respectively; and pET28a‐*nisP* for expression of protease NisP.

**Figure 1:**
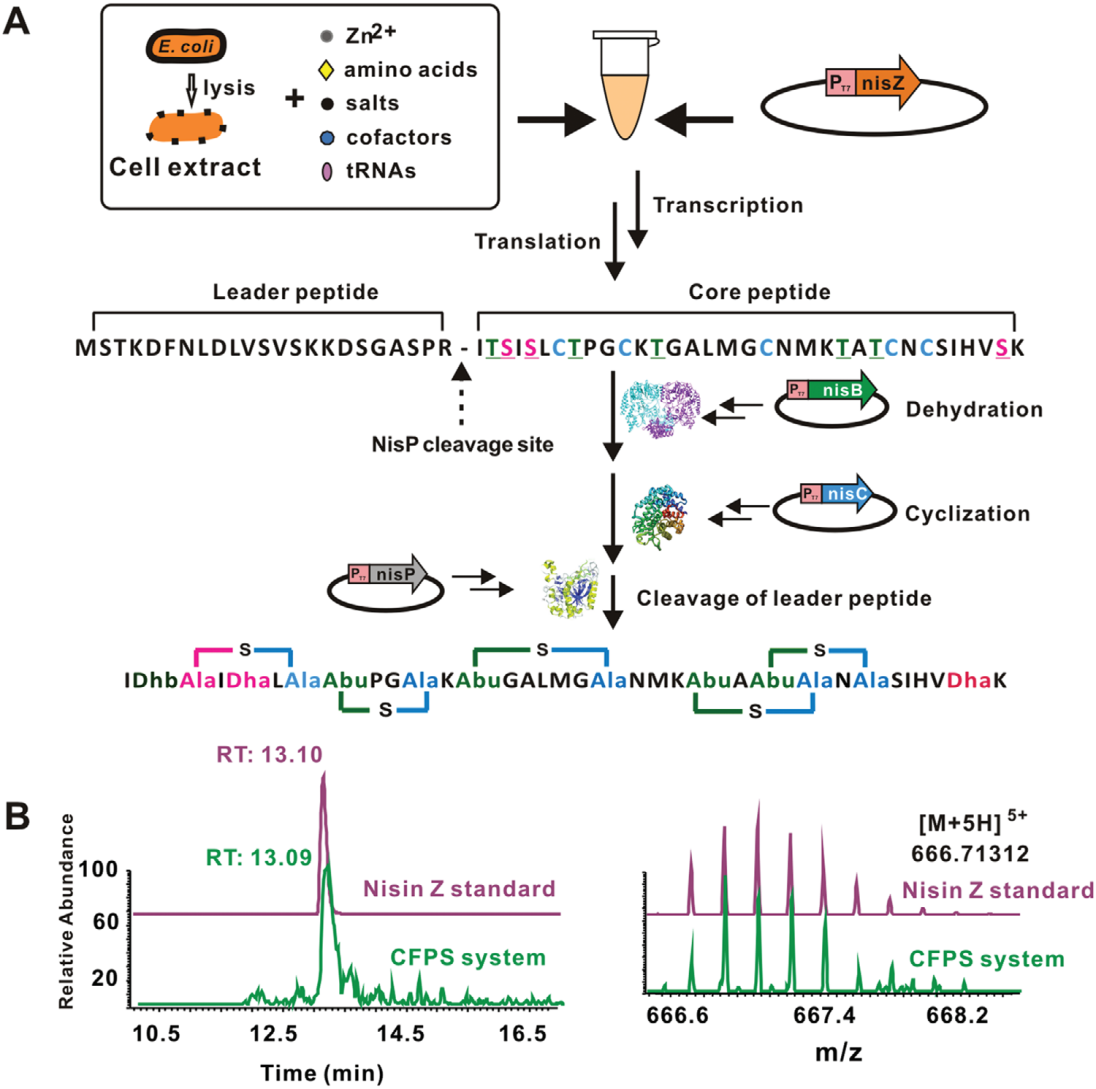
Reconstitution of nisin biosynthesis using the CFPS platform. A) Schematic illustration of nisin Z reconstitution in CFPS. Dha, dehydrolalanine; Dhb, dehydrobutyrine; Abu, 2‐aminobutyric acid. B) LC‐MS‐MS qualitative assay of nisin Z CFPS and standard. [M+5H]^5+^ ion of nisin Z (*m/z* = 666.71312) was used for ion monitoring.

We evaluated the performance of our CFPS platform for nisin biosynthesis. Each plasmid (0.5 nmol L^−1^) (pJL1‐*nisZ*, pET28a‐*nisB*, pET28a‐*nisC*, and pET28a‐*nisP*) was added into the CFPS platform and incubated for 6 h (**Figure** **1**A). The 400 µL of reaction mixture was concentrated to 20 µL and the concentrated mixture was then detected by tandem mass spectrometry (LC‐MS‐MS). The results confirmed that fully modified nisin Z was produced in the CFPS mixture (Figure 1B). The concentrated mixture was then used for antibacterial bioassays using the agar diffusion method, which revealed an obvious zone of inhibition on a *Micrococcus luteus* plate (Figure S3A, Supporting Information). This result confirmed that nisin Z synthesized by the CFPS platform was biologically active.

### 2.2 Optimization of the Nisin CFPS Platform

Although bioactive nisin was synthesized using the initial CFPS platform, the efficiency was low. Therefore, to enhance the efficiency of CFPS for synthesizing nisin, we used western blotting to analyze the accumulation of nisin biosynthetic enzymes and identified the rate‐limiting step of the initial nisin CFPS platform. We detected a single hybridization band at the expected size of the precursor peptides or modified precursor peptides (Figure S3B, Supporting Information), indicating that the other three enzymes responsible for nisin PTM (NisB and NisC) and leader peptide cleavage (NisP) were poorly expressed. Accordingly, his6‐tagged NisB, NisC, and NisPs (truncated NisP)^[^ ^17^ ^]^ were overexpressed in *E. coli* and purified. The rate‐limiting step was then investigated by systematically replacing each plasmid with 500 nmol L^−1^ of the corresponding purified protein. The substitutions of NisB and NisC significantly increased the titer of nisin Z (quantitative unit IU, 40 IU = 1 µg nisin);^[^ ^19^ ^]^ whereas the replacement of pET28a‐*nisP* with NisPs had minimal effects on nisin production (Figure S3C, Supporting Information). These results indicate that the dehydration and cyclization of the precursor peptide, controlled by NisB and NisC, serves as the rate‐limiting steps in the nisin PTM process. Moreover, the cleavage of the leader peptide did not act as a bottleneck.

**Figure 2:**
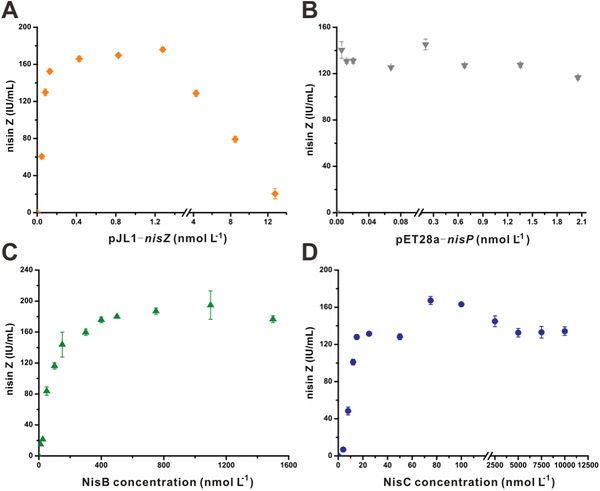
Optimization of nisin biosynthesis using the CFPS platform. A) Titration of pJL1‐*nisZ* involved in the nisin biosynthesis machinery (*n* = 3). B) Titration of pET28a‐*nisP* involved in the nisin biosynthesis machinery (*n* = 3). C) Titration of NisB involved in the nisin biosynthesis machinery (*n* = 3). D) Titration of NisC involved in the nisin biosynthesis machinery (*n* = 3). The data represented mean ± SD. 40 IU = 1 µg nisin.

We used systematic titration to study the optimal concentration of each component in the nisin biosynthetic pathway using the CFPS platform. First, concentrations of the precursor peptide gene encoded by plasmid pJL1‐*nisZ* were examined by varying the concentration, while fixing the concentrations of NisB and NisC to 0.5 µmol L^−1^ and pET28a‐*nisP* to 0.5 nmol L^−1^. The highest level of nisin Z production was detected at concentrations of pJL1‐*nisZ* of 0.4–1.3 nmol L^−1^ (**Figure** **2**A). A similar titration for pET28a‐*nisP* was applied to examine the effect of *nisP* concentration on the CFPS platform. Consistent with the results of our previous 500 nmol L^−1^ NisPs replacement experiments, the concentration of pET28a‐*nisP* did not appreciably influence nisin production (Figure 2B). Based on the *nisZ* and *nisP* titration studies, the optimal concentrations were determined to be 1.3 and 0.1 nmol L^−1^, respectively.

We next investigated the two enzymes responsible for nisin PTMs. In these assays, concentrations of pJL1‐*nisZ* and pET28a‐*nisP* were set to 1.3 and 0.1 nmol L^−1^, respectively. When testing the dehydration reaction, NisC was set to 500 nmol L^−1^, and the concentration of NisB was varied from 10 to 1500 nmol L^−1^. The titer of active nisin Z improved dramatically (from 15.1 ± 0.6 to 187.07 ± 4.1 IU mL^−1^) when the concentration of NisB increased from 10 to 800 nmol L^−1^ (saturation occurred at concentrations above 1000 nmol L^−1^ NisB; Figure 2C). Thus, increasing the concentration of NisB over a larger range would help increase nisin production. Moreover, a high NisB concentration did not inhibit nisin Z production. These results confirm that overexpression of *nisB* contributes substantially to nisin overproduction. Further, when the NisB concentration was 500 nmol L^−1^ and the NisC concentration was varied from 1 to 80 nmol L^−1^, the titer of active nisin Z ranged from 6.9 ± 2.0 to 167.3 ± 4.4 IU mL^−1^; a higher NisC concentration (from 100 to 10000 nmol L^−1^) did not appreciably influence nisin Z production (Figure 2D).

The concentrations of pJL1‐*nisZ* and pET28a‐*nisP* in the optimized cell‐free platform were low, and NisB and NisC were added as purified proteins, which eliminates the possibility of transcription and translation of PTM enzymes. To further prove that this system would not be hindered by interference from imbalanced transcription and translation, we added 100 ng of the pJL1‐*sfGFP* plasmid into the optimized cell‐free platform use for the synthesis of nisin Z for 6 h, and the results showed that sfGFP could be synthesized successfully, although the titer was lower than that produced in the system without nisin Z synthesis (Figure S4, Supporting Information). This result confirmed that the substrate and energy used in our system for transcription and translation were sufficient. Overall, the nisin titer of CFPS was increased by more than 30‐fold (from <5 to ≈180 IU mL^−1^) following replacement of nisin PTM enzyme, which is close to the highest nisin cell‐free synthesis titer reported to date (≈200 IU mL^−1^)^[^ ^14^ ^]^ that was achieved by synthesizing mNisA in a commercialized cell‐free synthesis kit followed by trypsin digestion to form the active nisin Z in vitro. This comparison further supported that the efficiency of our cell‐free platform may reach the limit of current cell‐free technology. The optimized CFPS platform was therefore used in subsequent experiments.

### 2.3 Use of the CFPS Platform for Rapid Genome Mining of Novel Lanthipeptides

In a previous genome mining study, the core peptide of a potential lanthipeptide was fused to the nisin leader peptide to form a hybrid precursor peptide, which were then coexpressed with *nisBC* and *nisT* (transporter) in *L. lactis*. The leader peptides of mLanAs were removed by NisP or trypsin in vitro to form bioactive lanthipeptides. Five novel lanthipeptides similar to nisin with antibacterial activity were identified in this way.^[^ ^5^ ^]^ Here, we explored the use of our CFPS platform for mining potential lanthipeptides (nisin analogs) with antibacterial activity using a similar strategy.

**Figure 3:**
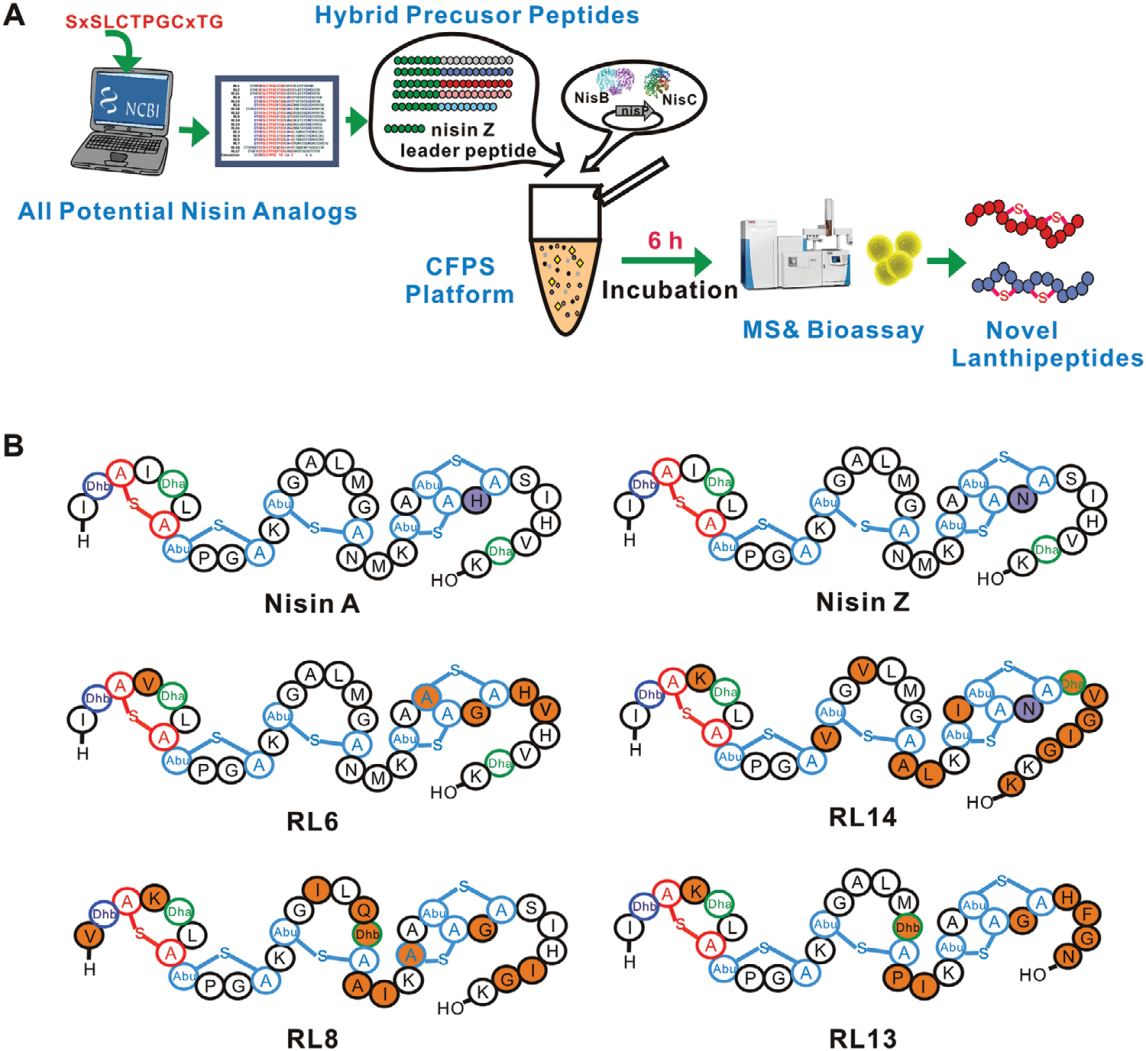
Rapid genome mining for novel lanthipeptides in CFPS. A) Schematic illustration of rapid genome mining for novel lanthipeptides using the CFPS system. B) Nisin and novel nisin analogs. Dha, dehydrolalanine; Dhb, dehydrobutyrine; Abu, 2‐aminobutyric acid.

We designed a rapid genome mining scheme (**Figure** **3**A). All proteins that were 40–80 amino acids in length were selected from the NCBI database (accessed June 2018). The resulting number of proteins was over two million. The sequence “SxSLCTPGCxTG” (where x denotes an arbitrary residue) was used to retrieve all potential nisin analogs. A total of 210 analogs were identified. We then excluded known lanthipeptides via sequence alignment in the BAGLE4 database^[^ ^20^ ^]^ and identified 18 potential lanthipeptides (RL1‐RL18; **Table** **1**), the core peptides of which were subsequently linked to the nisin leader peptide by gene synthesis to form hybrid precursor peptides (Table S1, Supporting Information).

**Table 1:** Identified nisin analog candidates

Sample	Core peptide	Modification	Anti‐*M. luteus*	Source
RL1^a)^	ITVRSKSLCTPGCITGPLRT CYLCFPTHVNC	(7)	N	*Bacillus velezensis*
RL2^a)^	ITWKSESLC TPGCVTGVLQT CFLQTIT CNCKISK	(9)	N	*Bacillus subtilis*/*Bacillus licheniformis*
RL3	VT SKSLC TPGCITGILMCLTQNS CVS CNS CIKC	(9)	N	*Bacillus thermoamylovorans*
RL4	IT SKSLC TPGCVTGILMT CPVQTAT CGCQITGK	6,7 (9)	N	*Blautia coccoides*
RL5	IT SKSLC TPGCITGILMCLTQNS CVS CNS CIRC	(9)	N	*Parageobacillus thermantarcticus*/*Anoxybacillus ayderensis*
RL6	IT SVSLC TPGCKTGALMGCNMKTAS CGCHVHVSK	6‐8 (8)	**Y**	*Leuconostoc gelidum*
RL7	IT SVSLC TPGCVTGVLMCPGNTIS CNGHC SIHITG	(9)	N	*Streptococcus pneumoniae*
RL8	VT SKSLC TPGCKTGILQT CAIKSAT CGC SIHIGK	6‐8 (9)	Y	*Bacillus cereus*
RL9	VT SKSLC TPGCITGVLMCLTQNS CVS CNS CIKC	(9)	N	*Aneurinibacillus sp*. XH2
RL10^a)^	ITVKIT SYSLC TPGCKTGALMGC TMKTAS CGCHVHISK	6‐9 (10)	N	*Lactobacillus salivarius*
RL11^a)^	ITWKSESLC TPGCITGVLQT CFLQTIT CNCHISK	(9)	N	*Bacillus nakamurai*
RL12	IT SYSLC TPGCITGVLMGCHIQSIGCNVHVHVSK	(7)	N	*Streptococcus salivarius*
RL13	IT SKSLC TPGCKTGALMT CPIKTAT CGCHFGN	6,7 (8)	**Y**	*Streptococcus equinus*
RL14	IT SKSLC TPGCVTGVLMGCALKTIT CNC SVGIGKK	5,6 (8)	**Y**	*Enterococcus rotai*/*Enterococcus moraviensis*
RL15	IT SKSLC TPGCVTGLLMGCAGS SAT CNC SVHVG	(9)	N	*Pseudobutyrivibrio sp*. UC1225/sp. 49
RL16	IT SKSLC TPGCVTGVLMGCNNKTAT CNC SVHVG	(8)	N	*Pseudobutyrivibrio sp*. UC1225/sp. 49
RL17^a)^	ITQFKSISLC TPGCPTGILMGCHKCPSGSDTVYTK	(9)	N	*Pseudobutyrivibrio sp*. UC1225/sp. 49
RL18	IT SPQIT SVSLC TPGCQTGFLACFSQACNPTGGCKISK	(10)	N	*Thermostaphylospora chromogena*
Bagelicin	VT SISLC TPGCKTGILMT CAIKTAT CGCHF	6‐8 (8)	**Y**	*Streptococcus suis* R61

a) represents the addition of I/IT to the N‐terminal of the core peptide to become a cleavable site recognized by NisP. “Modification” refers to the number of dehydrated residues based on the LC‐MS‐MS observed. The total number of serines and threonines in the peptide are presented in parentheses. Cysteines are depicted as blue, serines are purple and threonines are green.

These hybrid precursor peptides were then cloned into the pJL1 plasmid. Bagelicin, a nisin analog with antibacterial activity that was identified using a previously described in vivo screening system,^[^ ^5^ ^]^ was chosen as the positive control for validation. The pJL1‐*nisZ* plasmid in our CFPS platform was replaced with the hybrid precursor peptide plasmids (pRL1‐pRL18 and pJL1‐bagelicin). After incubation for 6 h, the bagelicin CFPS mixture (positive control) was analyzed by LC‐MS‐MS and bioassays. The results indicate that the Thr and Ser residues in bagelicin were dehydrated and that the bagelicin CFPS mixture contained anti‐*M. luteus* activity (Table 1), demonstrating that our CFPS platform can complete the previous work of mining novel lanthipeptides using in vivo methods.^[^ ^5^ ^]^ Six candidate lanthipeptides were dehydrated based on LC‐MS‐MS detection, among which four displayed anti‐*M. luteus* activity (Table 1).

The CFPS system is for quick screening of potential antimicrobial lanthipeptides, and we then purified enough compounds of the screened nisin analogs for structure identification and activity assays. We prepared mLanAs for the novel lanthipeptides (RL6, RL8, RL13, and RL14) in *E. coli* (Table S2, Supporting Information) and removed their leader peptides in vitro, as it is more economical to mass produce targeted lanthipeptides using this previously reported method^[^ ^4^ ^]^ rather than to use CFPS to mass produce lanthipeptides. Nisin and most lanthipeptides are not pure products in the commercially available and standard products, they are a mixture of different dehydrating ingredients resulting from incomplete NisB catalysis. Therefore, we verified that the decisive antibacterial component in the currently commercially available nisin is the eightfold dehydrated component (Figure S5, Supporting Information). The structure of nisin was determined using its most effective antibacterial component (eightfold dehydrated component), and the eight‐fold dehydrated components of RL6, RL8, RL13, and RL14 were also used to determine their structure (Figure S6, Supporting Information). We treated the novel lanthipeptides with the thiol‐alkylating reagent *N*‐ethylmaleimide (NEM) to sequester any noncyclic Cys residues, and evaluated ring topology using LC‐MS (Figure S7, Supporting Information). The results show that all five thioester rings in their core peptides were formed in mature lanthipeptides; their structure are shown in Figure 3B. We quantified these four novel lanthipeptides using the purified eight‐fold dehydrated component of nisin as a standard. The concentration of their most effective antimicrobial components (eight‐fold dehydrated components) was determined, and the antibacterial activity of novel lanthipeptides against *M. luteus* and clinical pathogenic strains of *Enterococcus faecalis, Staphylococcus aureus*, and methicillin‐resistant *S. aureus* (MRSA) was then tested. All four tested lanthipeptides exhibited antibacterial activity against these bacteria, with RL14 outperforming nisin against *M. luteus* and *E. faecalis* (Table S3, Supporting Information).

### 2.4 Screening of Lanthipeptides Activity Using the CFPS Platform

Next, we evaluated the performance of the CFPS platform for library screening, an application that has been reported using in vivo methods.^[^ ^6^, ^7^ ^]^ We screened mutant lanthipeptides using the CFPS platform to extend the specific activity of nisin against gram‐positive bacteria to gram‐negative bacteria. The screening of mutant nisin with anti‐gram‐negative bacteria has been reported by the knockout of *nisA* in a producing nisin A *L. lactis* and the introduction of a plasmid library harboring *nisA* mutants. Several nisin mutants (including S29A) with anti‐*E. coli* activity were identified using this approach.^[^ ^21^ ^]^

**Figure 4:**
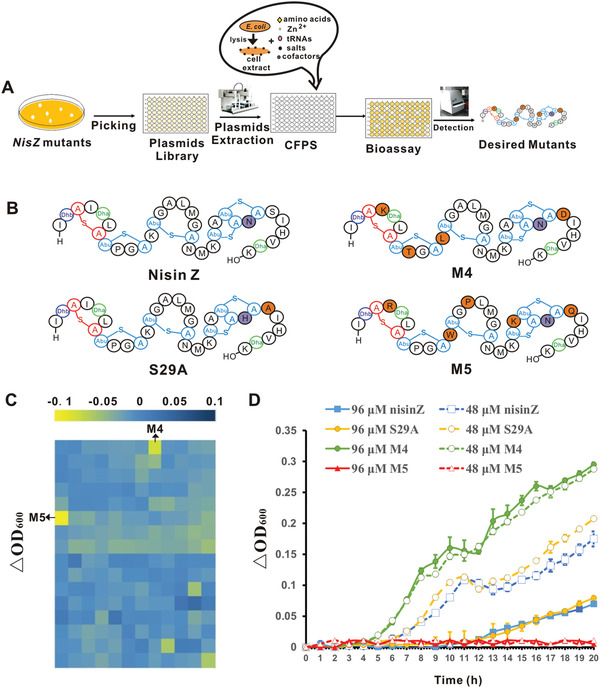
Screening of nisin mutants using the CFPS system. A) Schematic illustration of the screening for nisin mutants. B) Nisin and nisin mutants. Dha, dehydrolalanine; Dhb, dehydrobutyrine; Abu, 2‐aminobutyric acid. C) Heat‐map of antimicrobial activity of nisin mutants against *E. coli* in the second round of screening. After adding the CFPS mixture to *E. coli* and incubating for 8 h, the ΔOD_600_ value represents the OD_600_ of *E. coli* with added nisin mutant CFPS mixture minus the OD_600_ (control group) of *E. coli* with nisin CFPS mixture in the same plate. Yellow areas represent an inhibitory effect on *E. coli*. D) OD_600_ of *E. coli* DH5*α* in LB medium with 320 µmol L^−1^ EDTA after treatment with nisin and nisin mutants (*n* = 3). The ΔOD_600_ value represents the OD_600_ of *E. coli* with lanthipeptide minus the OD_600_ of blank LB medium with the same concentration of lanthipeptide. The test lanthipeptide is a mixture of different dehydration molecules, quantification of lanthipeptides was performed using the eightfold dehydrated molecules. The data represented mean ± SD.

We aligned the reported nisin analogs (nisin A, nisin Z, nisin F,^[^ ^22^ ^]^ nisin Q,^[^ ^23^ ^]^ and nisin U^[^ ^24^ ^]^) and selected five amino acid residues in nonconserved regions (positions 4, 12, 15, 24, and 29 of nisin Z) for saturation mutagenesis to form a nisin mutants library. The library was constructed with mixed plasmids, and the mixed plasmids library was transformed to *E. coli* so that the plasmids can be separated. Randomly selected monoclonal colonies were inoculated in 96‐well plates and cultured at 37 °C for 16 h. Cultured *E. coli* was lysed to obtain plasmid‐containing cell extracts, and these extracts were added directly to our CFPS system. The nisin mutants were synthesized using our optimized CFPS platform on microplates with 6‐h incubation periods. The CFPS reaction mixture was then co‐cultured with *E. coli* in 96‐well plates, and the OD_600_ of the culture was determined after 8 h (**Figure** **4**A). Cultures with lower OD_600_ in the same batch of experiments were considered to have the potential to inhibit the growth of *E. coli*. To improve the accuracy of the screening, we conducted two rounds. In the first round, ≈190 candidates with the potential to inhibit the growth of *E. coli* were selected from ≈3000 mutants. The mutants among these candidates that showed greater inhibition of *E. coli* than nisin were selected in the second round of screening. Two mutants exhibited stronger inhibitory effects against *E. coli* than nisin in the second round of screening (Figure 4C). By sequencing the two plasmids, the M4 mutant was identified and named based on the four mutations in its core peptide (I4K, P9T, K12L, and S29D). The mutant in position 9 may have been caused by an unwanted mutation generated during synthesis of the mutation library, or may have resulted from mutations acquired during replication in *E. coli*. Additionally, the M5 mutant was identified and named based on the five mutations in its core peptide (I4R, K12W, A15P, A24K, and S29Q).

Next, to verify the reliability of the CFPS platform for screening, abundant lanthipeptides are required to characterize the structure and anti‐*E. coli* ability of the mutants. We purified M4, M5, and S29A using the method described for the expression of mLanAs in *E. coli* (Table S2, Supporting Information), and removed the leader peptides in vitro. The structures were identified by LC‐MS‐MS (Figure 4B; Figure S6, Supporting Information) and the thioester rings were detected by NEM reaction (Figure S7, Supporting Information). Nisin exhibited observable inhibition against some strains of *E. coli*;^[^ ^21^, ^25^ ^]^ however, these strains were not available in our laboratory. To ensure comparable conditions to previous reports, DH5*α* with 320 µmol L^−1^ EDTA was used based on the apparent growth inhibition of *E. coli* with different concentrations of nisin (Figure S8, Supporting Information). We generated the *E. coli* DH5*α* growth curve using M4, M5, S29A, or nisin Z with 320 µmol L^−1^ EDTA and found that M5 inhibited growth more effectively against gram‐negative *E. coli* than did nisin Z or S29A in this condition (Figure 4D). These results suggest that our novel CFPS platform is effective in screening for lanthipeptides.

### 2.5 Targeted Metabolic Engineering for Nisin and Nisin Analog Overproduction In Vivo

**Figure 5:**
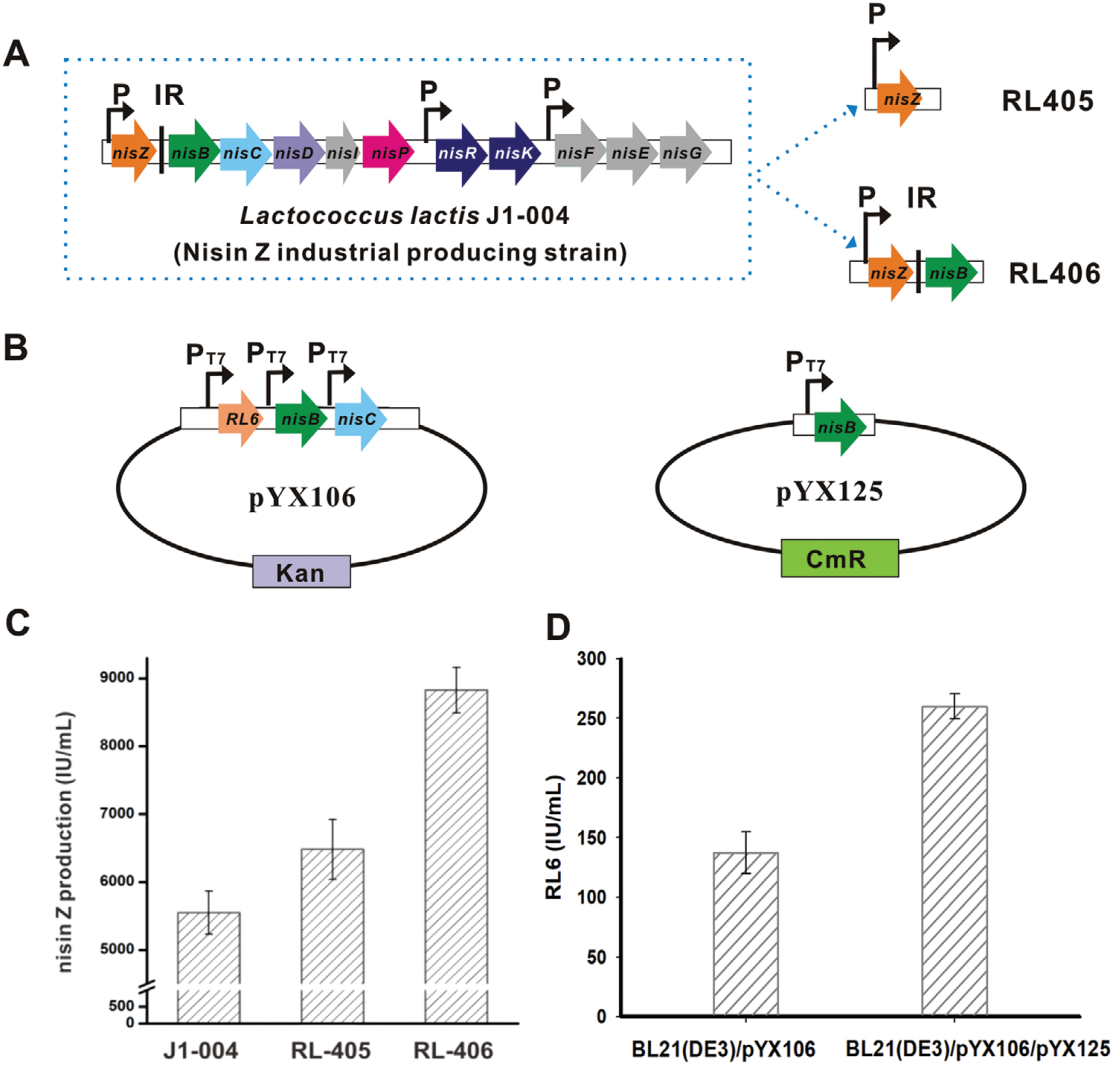
CFPS platform guides the overproduction of lanthipeptides. A) Original nisin biosynthesis gene cluster in *L. lactis* J1‐004 and construction of the overexpression operons used in this study. B) Plasmids for the expression of the modified RL6 precursor peptide in *E. coli*. C) Production of nisin in industrial strains and the individual engineered strain after 16 h of fed‐batch fermentation (*n* = 3). D) Novel lanthipeptide RL6 production in different *E. coli* strains (*n* = 3). These two *E. coli* strains were fermented to overexpress modified RL6 precursor peptides and digested with trypsin in vitro. Nisin was used as the quantitative standard. The data represented mean ± SD. 40 IU = 1 µg nisin.

We have learned from the process of optimizing the CFPS platform that increasing NisB significantly increased nisin Z production. We next evaluated this strategy using industrial *L. lactis* strains for nisin Z overproduction. The industrial nisin Z‐producing strain is difficult to manipulate genetically because it has been used for mutations for decades, reducing its ability to accept foreign DNA. Hence, the targeted genetic procedure employed is critical when working with this industrial strain because it can only be manipulated once. To verify whether the overexpression of *nisB* in vivo could increase nisin production, RL405 for overexpression of *nisZ* and RL406 for co‐overexpression of *nisZ* and *nisB* were constructed (**Figure** **5**A). The results (Figure 5C) show the industrial strain J1‐004 produced 5549.0 ± 316.3 IU mL^−1^ nisin Z after 16 h of fed‐batch fermentation, whereas RL405 produced 6479.7 ± 443.9 IU mL^−1^ nisin Z under the same fermentation conditions, which was 16.8% higher than that of J1‐004. However, the *nisB* overexpressed strain, RL406, exhibited an increase in nisin Z production to 8828 ± 336.2 IU mL^−1^, representing a nearly 60% increase over that of the industrial strain J1‐004 and a 36.2% improvement to RL405. This result further demonstrated that the overexpression of *nisB* contributed substantially to nisin overproduction. We further analyzed the nisin biosynthesis proteins in the engineered *L. lactis* strains by a targeted proteomics approach. NisZ was detected in RL405 and both NisZ and NisB were detected in RL406, which shows that *nisZ* and/or *nisB* were overexpressed as designed (Figure S9, Supporting Information).

To investigate whether the overexpression of *nisB* could also increase the titer of nisin analogs in a heterologous expression host, *E. coli* BL21(DE3)/pYX106 (Table S2, Supporting Information) was constructed according to a previously described method^[^ ^4^ ^]^ with *lanA* of RL6, *nisB*, and *nisC* coexpressed in pYX106 for the production of mLanA of RL6 (mRL6) (Figure 5B). BL21(DE3)/pYX106/pYX125 (Table S2, Supporting Information) was constructed for the overproduction of mRL6 with an extra copy of *nisB* expression in pYX125 besides pYX106 (Figure 5B). The parallel fermentation of these two strains resulted in no obvious differences in OD_600_ values, indicating that the overexpression of *nisB* had no effect on the growth of *E. coli*. After cleavage of the leader peptide in vitro by trypsin, the *nisB* overproduction strain BL21(DE3)/pYX106/pYX125 produced RL6 at 260.1 ± 10.5 IU mL^−1^, whereas the control strain BL21(DE3)/pYX106 produced 137.5 ± 17.5 IU mL^−1^. The overexpression of *nisB* increased the titer by 89.2% in *E. coli* (Figure 5D), confirming *nisB* is a universal key determinant for nisin analogs lanthipeptides overproduction. We can conclude from these results that the CFPS platform guided successful overproduction of lanthipeptides.

## 3 Conclusion

We have developed an optimized CFPS platform for the genome mining of nisin analogs, screening of mutants, and guiding lanthipeptides overproduction in vivo. The functionality of this platform was verified in that all nisin analogs with bactericidal effects were mined using our CFPS platform in a single day, one of which (RL14) exhibited stronger antibacterial activity than that of nisin. Moreover, a nisin mutant (M5) with higher activity against gram‐negative bacteria (EDTA‐treated) than nisin was screened using this platform; and a 60% nisin Z increase in an industrial host and an 89.2% increase in nisin analogs in a heterologous expression host were observed.

The first advantage of our CFPS platform for lanthipeptide research is efficiency. Although we used purified nisin PTM enzyme proteins in the CFPS platform for improved efficiency, these enzymes can be purified in abundance and stored at −80 °C for an extended period of time, allowing lanthipeptides to be synthesized from DNA templates in 6 h. The second advantage of our platform is that the production of lanthipeptides is not dependent on cell growth. To ensure that it has the advantage of screening for the bacteriostatic activity of lanthipeptides, we developed the process of screening lanthipeptide with anti‐*E. coli* (EDTA‐treated) activity. However, by changing the indicator strain, the platform can be used for screening other bacteriostatic lanthipeptides as well. The third advantage is that the reaction volume is 8–10 µL, and the reaction mixture can be processed for use in bioassays and MS detection (after desalting) without the requirement for complex purification processes. It is, therefore, reasonable to suggest that the CFPS platform can perform high‐throughput screening work. The fourth advantage is the rapid and accurate identification of the rate‐limiting step, regardless of the complex regulatory mechanisms in the cell.

We provide an effective method for the cell‐free production of lanthipeptides, with applications for the biosynthesis of other RiPPs. The direct synthesis of lanthipeptides using DNA templates is less efficient than synthesis using purified enzymes; however, the development of cell‐free systems with higher titers,^[^ ^26^ ^]^ lower costs,^[^ ^27^ ^]^ and simpler preparation methods^[^ ^28^ ^]^ is an active area of research. These studies will contribute to increasing the efficiency of directly synthesized RiPPs using DNA templates. Moreover, addition of extra tRNA^Glu^, optimization of DNA codon, ion concentrations, or redox environment may also improve the efficiency of CFPS and allow it to play a more prominent role in RiPPs research.

In the CFPS platform, we used PTM enzymes (NisB and NisC) with clear catalyzed machinery to modify other potential lanthipeptides (using hybrid precursor peptides). Although novel lanthipeptides may not share the same natural structure as their original structure in vivo, this method represents a general and rapid approach for genome mining for potential RiPPs. More importantly, owing to the diversity of PTMs, different types of PTM enzymes can be applied to generate novel RiPPs in a combinatorial biosynthesis manner.^[^ ^29^ ^]^ Owing to the robustness and flexibility of CFPS, different PTM enzymes can readily be combined in this cell‐free system to achieve novel RiPPs with diverse bioactivities.

CFPS can be easily developed as an automated high‐throughput screening platform. The Freemont group at ICL has developed a rapid automated method using a cell‐free platform to quantify a series of ribosome‐binding site mutants and uncharacterized endogenous constitutive and inducible promoters to characterize new DNA components in nonmodel bacteria.^[^ ^30^ ^]^ It is, therefore, reasonable to suggest that combined automated high‐throughput platforms containing CFPS with different microbes, such as *Streptomyces* and cyanobacteria that possess many RiPP gene clusters, will accelerate the rate of discovery for novel RiPPs because their cell extracts may contain specific components related to the synthesis of RiPPs. Overall, our research extends the use of cell‐free systems to address the issue facing lanthipeptide overproduction and novel compound discovery, while providing the possibility for development of CFPS platforms for other RiPPs studies.

## 4 Experimental Section

##### Strains

The strains used in this study are listed in Table S2 (Supporting Information). *E. coli* was grown in lysogeny broth (LB) with appropriate antibiotics (Kan: 50 µg mL^−1^, CmR: 34 µg mL^−1^) at 37 °C. *L. lactis* was grown in M‐17 (Oxoid, Thermo Fisher Scientific, Waltham, MA, USA) supplemented with 0.5% d‐glucose (GM‐17) at 30 °C with appropriate antibiotics (EmR: 5 µg mL^−1^). *M. luteus* was grown in bioassay medium with 1.2% tryptone, 0.75% yeast extract, 0.75% NaCl, 0.3% NaH_2_PO_4_, and 0.75% d‐glucose at 37 °C. *E. faecalis*, *S. aureus*, and MRSA were grown in Mueller–Hinton broth (MHB; BD Difco, Franklin Lakes, NJ, USA) at 37 °C.

##### Plasmids Construction

The primers and plasmids used for strain construction are listed in Tables S4 and S5 (Supporting Information), respectively. In general, the primers used to construct the relevant plasmids were named after the plasmid, and the corresponding restriction enzyme sites were designed on the primers. For instance, pJL1‐nisZ‐F and pJL1‐nisZ‐R were used for *nisZ* amplification and the PCR fragment cloned into the *Nde*I and *Bam*HI sites of pJL1^[^ ^31^ ^]^ to yield pJL1‐*nisZ*. All fragments obtained by polymerase chain reaction were gel‐purified using a DNA gel extraction kit (Axygen, Corning, NY, USA) according to manufacturer's instructions, before cloning.

For the biosynthesis of nisin and other lanthipeptides in the CFPS system, genomic DNA from *L. lactis* was obtained using the Blood & Cell Culture DNA mini kit (QIAGEN, Hilden, Germany) following the manufacturer's instructions. *nisZ* was amplified by PCR from *L. lactic* J1‐004, subcloned to pJL1,^[^ ^31^ ^]^ and designated as pJL1‐*nisZ*. *nisB*, *nisC, nisP*, and *nisPs* were individually amplified by PCR from *L. lactic* J1‐004 and cloned to pET28a(+) (Novagen, Darmstadt, Germany) to yield pET28a‐*nisB*, pET28a‐*nisC*, pET28a‐*nisP*, and pET28a‐*nisPs*, respectively. Each hybrid lanthipeptide RL1‐RL18 (see Table S1, Supporting Information for sequences) was amplified using pRL‐F as the general forward primer and pRLX‐R as the reverse primer (X represents a specific plasmid; Table S4, Supporting Information). For example, the reverse primer for pRL1 is pRL1‐R, followed by the PCR fragment cloned into the *Nde*I and *Bam*HI sites of pJL1‐*nisZ*, respectively, yielding pRL1‐pRL18.

For the expression of mLanAs in *E. coli*, several plasmids were constructed. An ≈3 kb *Nde*I/*Kpn*I fragment containing *nisB* was inserted into the corresponding sites of the plasmid pRSFDuet‐1 and yielded plasmid pYZ82. In the plasmid pYZ85, the *lanA* of RL6 was inserted between the *Bam*HI and *Eco*RI sites of the plasmid pYZ82, which was constructed to express the *lanA* (RL6) gene with a His6‐tag and *nisB*. Similarly, pYZ86, pYZ87, pYZ89, pYZ90, and pYZ91 were constructed by inserting the *lanA* (RL8, S29A, M5, M4, and NisZ) genes between the *Bam*HI and *Eco*RI sites of the plasmid pYZ82, respectively. Five plasmids, pYZ92, pYZ93, pYZ95, pYZ96, pYZ97, and pYZ99, were constructed to replace the His6‐tag in pYZ90, pYZ89, pYZ85, pYZ86, pYZ87, and pYZ91 with the sumo‐tag. Primers Sumo‐F and Sumo‐R were used to amplify the sumo‐tag fragment from pSUMO. The plasmid pYZ81 for the expression of *nisC* under the control of T7 promoter used an ≈1.25 kb *Kpn*I/*Xho*I fragment inserted into the corresponding sites of the plasmid pACYCDuet.

For the expression of the mLanAs in *E. coli* with *nisB* overexpression, several plasmids were constructed. The 1.5‐kb *Xho*I/*Xho*I fragment that contained the T7 promoter and *nisC* was inserted into the corresponding sites of the plasmid pYZ85, yielding pYX106. The plasmid pYX125 for the expression of *nisB* under the control of the T7 promoter used an ≈3 kb *Kpn*I/*Xho*I fragment inserted into the corresponding sites of the plasmid pACYCDuet. The plasmid pYX105 was constructed to produce a modified sumo‐tag S29A precursor peptide (coexpression of S29A, *nisB* and *nisC*). An ≈0.2 kb *Bam*HI/*Eco*RI fragment of a different *lanA* was inserted into the corresponding sites of the plasmid pYX105, yielding pYX122 (sumo‐tag‐mRL13) and pYX123 (sumo‐tag‐mRL14). For the plasmid pYX126, *nisB* under the control of the T7 promoter was inserted into *Xho*I site of the plasmid pYZ81 for the expression of *nisB* and *nisC*.

For overexpression of lanthipeptides in *L. lactis*, two plasmids were constructed via the Gibson assembling method.^[^ ^32^ ^]^ The plasmid pRL415 overexpressing *nisZ* under the control of Pnis promoters was constructed. Primers pRL415‐F and pRL415‐R were used for the amplification of the Pnis‐*nisZ* operon from *L. lactic* J1‐004. Primers pRL415‐VF and pRL415‐VR were used for the amplification of the pMG36e backbone. The plasmid pRL423 for the overexpression of *nisZ* and *nisB* under the control of the Pnis promoter was constructed by coamplification in their original order in the *nisZ*BTCIP operon.^[^ ^33^ ^]^ Primers pRL423‐F and pRL423‐R were used for the amplification of *nisB* from *L. lactic* J1‐004. Primers pRL423‐VF and pRL423‐VR were used for amplification from pRL415.

##### NisB, NisC, and NisPs Purification

The protein NisPs was purified according to a previously described method.^[^ ^17^ ^]^ NisB and NisC were overexpressed and purified according to the previously described NisB purification method.^[^ ^34^ ^]^ Briefly, *E. coli* BL21 (DE3) cells were transformed with pET28a‐*nisB* and pET28a‐*nisC*. BL21 Rosetta (DE3) was transformed with pET28a‐*nisPs*. Several colony transformants were then grown in 50 mL of media supplemented with 50 µg mL^−1^ Kan at 37 °C overnight. A 1% inoculation of a 2 L of LB‐antibiotic culture was grown aerobically at 37 °C until OD_600_ reached 0.6–0.8. Then, 0.1 mmol L^−1^ IPTG was added for NisPs induction and further grown for 3 h at 37 °C. NisB and NisC cells were cooled to 18 °C and IPTG was added to a final concentration of 0.5 mmol L^−1^ (NisB) or 0.2 mmol L^−1^ (NisC) and further grown for 20 h. For NisC overexpression, an additional 100 µmol L^−1^ ZnCl_2_ was added to ensure enzymatic activity of NisC. After further growth, cells were harvested by centrifugation at 5000 × *g* for 20 min at 4 °C and resuspended in buffer A (20 mmol L^−1^ Tris, pH 7.6, 500 mmol L^−1^ NaCl, 10% glycerol). The cell suspension was lysed by homogenization at a variable pressure of 10 000–15 000 psig and centrifuged at 25000 × *g* for 1 h at 4 °C. The Ni‐NTA column (GE Healthcare, Marlborough, MA, USA) was charged and washed with 2 column volumes (CV) of buffer A, and the filtered supernatant was applied to the column. The resin was washed with 2 CV each of buffers containing 0, 25, 50, 100, 200, and 500 mmol L^−1^ imidazole. The purified protein was concentrated using Amicon Ultra‐15 centrifugal filter devices (Millipore, Billerica, MA, USA) and the buffer was replaced with storage buffer (100 mmol L^−1^ phosphate buffer, 10% glycerol, pH 7.6) via the PD‐10 column (GE Healthcare). Protein concentrations were measured using a Pierce BCA protein assay kit (Thermo Fisher Scientific) according to manufacturer's instructions. Proteins were stored at −80 °C after flash freezing in liquid nitrogen.

##### CFPS Reactions

CFPS reactions were performed to synthesize lanthipeptides. The previously described crude extract‐based CFPS system^[^ ^18^, ^35^ ^]^ was used for in vitro transcription and translation and supplemented with essential components for lanthipeptide PTMs. In the preparation of *E. coli* BL21(DE3) cell extracts, 1 mmol L^−1^ isopropylthio‐*β*‐galactoside (IPTG) was added during mid‐log phase to induce T7 RNA polymerase overexpression, and cell lysate protein concentrations were measured using a Pierce BCA protein assay kit (Thermo Fisher Scientific). The reaction mixture for CFPS consisted of the following components in a final volume of 8– 400 µL containing 1.2 mmol L^−1^ ATP; 0.85 mmol L^−1^ each of GTP, UTP, and CTP; 34.0 µg mL^−1^ l‐5‐formyl‐5,6,7,8‐tetrahydrofolic acid (folinic acid); 170.0 µg mL^−1^ *E. coli* tRNA mixture; 130 mmol L^−1^ potassium glutamate; 10 mmol L^−1^ ammonium glutamate; 12 mmol L^−1^ magnesium glutamate; 2 mmol L^−1^ each of 20 natural amino acids; 0.33 mmol L^−1^ nicotinamide adenine dinucleotide (NAD); 0.27 mmol L^−1^ coenzyme‐A (CoA); 1.5 mmol L^−1^ spermidine; 1 mmol L^−1^ putrescine; 4 mmol L^−1^ sodium oxalate; 33 mmol L^−1^ phosphoenolpyruvate (PEP); 10 µmol L^−1^ ZnCl_2_ (for NisC cyclization), and 27% v/v cell extracts. For each reaction, plasmids, purified NisB, and purified NisC were added at various concentrations. The CFPS reactions were performed at 30 °C for 6 h. Reactions were terminated by incubation at 85 °C for 10 min, and precipitated proteins were pelleted by centrifugation at 10 000 × *g* for 5 min. The resulting supernatant was subjected to downstream analyses.

##### Qualitative and Quantitative Analyses by LC‐MS‐MS

Qualitative analyses of nisin Z and other lanthipeptides were performed using a previously described LC‐MS‐based method with modifications.^[^ ^36^ ^]^ Briefly, the supernatant of the CFPS reaction mixture or other solutions treated with trypsin were obtained after centrifugation at 10 000 × *g* for 10 min. Supernatants were desalted using Sep‐Pak Vac C18 cartridges (Waters, Milford, MA, USA) and subjected to LC‐MS‐MS. Chromatographic separation was performed using a Thermo Fisher Ultimate 3000 UPLC system equipped with a Thermo Fisher Hypersil GOLD C18 (2.1 × 100 mm, 3 µm) HPLC column; mobile phase A was H_2_O (0.1% formic acid) and mobile phase B was acetonitrile (ACN). The gradient program was (time, B%) 0 min, 10% B; 5 min, 10% B; 25 min, 95% B; 35 min, 95% B; 35.1 min, 10% B; 40 min, 10% B. The flow rate was 200 µL min^−1^. The column temperature was 35 °C and the injection volume was 10 µL. The sampler tray temperature was 8 °C. Detection was performed using a Thermo Fisher Q Exactive Orbitrap MS with an ESI source in positive ion mode. Instrument parameters were as follows: sheath gas set to 35; auxiliary gas set to 5 (arbitrary units); spray voltage 3.5 kV; capillary temperature 320 °C; probe heater temperature 250 °C. Full scan trigger dd‐MS_2_ mode was used for qualitative condition determination, and the settings were as follows: scan range 150–2000 Da; MS resolution 70 000; MS_2_ resolution 17 500; isolation window 1.4 m/z; CE 30; dynamic exclusion of 2 s.

For lanthipeptides quantification, full scan mode was used. A semipreparative HPLC was used to collect the eightfold dehydrated component of nisin (see Supporting Information) and then use this as a standard to quantify nisin analogs or nisin mutants. The collected eightfold dehydrated component of nisin was lyophilized and then used to generate a standard curve. Angiotensin II (10 ppb) was added as an internal standard to each concentration of eightfold dehydrated component of nisin. The ordinate axis of the standard curve corresponds to the concentration of the eight‐fold dehydrated component of nisin (linear range: 10–20 000 ppb), and the abscissa axis the ratio of the corresponding different concentrations of the eightfold dehydrated component of nisin to 10 ppb angiotensin II. Angiotensin II (10 ppb) was also added as an internal standard to tested samples, allowing the calculation of the ratio of eightfold dehydrated component of nisin‐like lanthipeptide to 10 ppb angiotensin II and the quantification of nisin analogs or nisin mutants. Additionally, 200 ppm BSA solution was used as a dilution reagent.

##### Agar Diffusion Assay

The activity levels of nisin and new lanthipeptides were determined by a previously described agar diffusion method, with minor modifications.^[^ ^37^ ^]^ Briefly, a stock nisin solution 1000 IU mL^−1^) was prepared by adding 50 mg of commercial nisin (10^6^ IU g^−1^; Sigma‐Aldrich, St. Louis, MO, USA) into 50 mL of sterile 0.02 mol L^−1^ HCl. Standard nisin solutions of 1000, 500, 250, 200, 100, 20, and 5 IU mL^−1^ were prepared using the stock solution and diluted with 0.02 mol L^−1^ HCl to construct a standard curve. The bioassay medium contained 1.2% tryptone, 0.75% yeast extract, 0.75% NaCl, 0.3% NaH_2_PO_4_, and 2% agar. After the addition of sterile 0.75% glucose and 0.5% Tween 20, the agar medium was cooled to 50°C and inoculated with 1.5% overnight culture of the indicator strain *M. luteus* NCIB 8166. The bioassay agar (25 mL) was aseptically poured into sterile Petri dishes, and several holes were made on each plate after solidification. Then, 2 µL of the CFPS mixture supernatant and an equal volume of the nisin Z standard solution were separately added to the holes. After incubation at 30 °C for 18 h, a standard curve of the nisin zone of inhibition versus units of the nisin standard solution was created by measuring the zone diameter using digital calipers (TAJIMA Tool Co., Ltd., Shanghai, China) horizontally and vertically. Nisin concentrations for each CFPS mixture were estimated.

##### In Silico Prediction and Selection of Nisin Analogs

Protein sequences for bacteria and fungi with lengths of 40–80 amino acid residues were retrieved from the NCBI database (accessed June 2018). The sequences containing the conserved motif of the core peptide (SxSLCTPGCxTG, where x denotes an arbitrary residue) were retained. The sequences were further reduced by applying the filter rule that if there are not LanBC‐like or LanM‐like proteins among the ten genes upstream or downstream of the 40–80 amino acid residues led to exclusion of the sequences. Finally, after the exclusion of known peptides using BAGEL4,^[^ ^20^ ^]^ 18 candidates were selected for experimental validation. These 18 sequences were adjusted to conform to the recognition site of NisP (if the N‐terminal amino acid residues of the core peptide sequence were not “IT” or “VT,” “I/IT” was added to the N‐terminus).

##### Overexpression and Purification of Modified Lanthipeptides Precursor Peptides

The overexpression and purification of his6‐tagged mLanAs were performed as described previously.^[^ ^4^ ^]^ Briefly, overnight cultures were grown from a single recombinant *E. coli* colony transformant and used as inoculum to grow 1.5 L of Terrific broth containing 50 mg L^−1^ Kan and 34 mg L^−1^ CmR at 37 °C until the OD_600_ reached 0.6–0.8. The incubation temperature was adjusted to 18 °C and the culture was induced with 0.5 mmol L^−1^ IPTG. The induced cells were shaken continuously at 18 °C for an additional 18–20 h. The cells were harvested by centrifugation (11 900 × *g* for 10 min; Beckman JLA‐10.500 rotor). The cell pellet was resuspended in 45 mL of start buffer (20 mmol L^−1^ Tris, pH 8.0, 500 mmol L^−1^ NaCl, 10% glycerol, containing a protease inhibitor cocktail from Roche Applied Science) and lysed by homogenization at variable pressures from 10 000–15 000 psig and centrifuged at 25 000 × *g* for 1 h at 4 °C. The supernatant was loaded onto a nickel affinity column pre‐equilibrated with start buffer. After loading, the column was washed with wash buffer (start buffer + 30 mmol L^−1^ imidazole). The peptide was eluted from the column using elution buffer (start buffer + 500 mmol L^−1^ imidazole), and the elution buffer with the targeted modified peptide was replaced with PB buffer using an Amicon Ultra centrifugal filter (Millipore). Trypsin (2.5%) was added to the peptide solution and digested for 4 h at 37 °C.

##### N‐Ethylmaleimide (NEM) Alkylation Assay of Cyclization

NEM alkylation assays were performed according to a previously reported method.^[^ ^6^ ^]^ Briefly, an aliquot of the protease‐digested peptide or full‐length peptide solution was diluted into a twofold volume of NEM alkylation buffer containing 500 mmol L^−1^ HEPES, 3 mmol L^−1^ NEM, 0.3 mmol L^−1^ Tris(2‐carboxyethyl)phosphine (TCEP) (pH 6.5). The reaction was incubated at 37 °C for 30 min in the dark and analyzed by LC‐MS‐MS. Species containing uncyclized free Cys residues that were alkylated were identified by a mass increase of 125 Da for each adduct.

##### Antibacterial Assays

MICs were determined according to a previously described method^[^ ^38^ ^]^ with minor modifications. Briefly, the test medium for clinical strain species was MHB broth (BD Difco), LB broth was used for *E. coli*, and bioassay medium containing 1.2% tryptone, 0.75% yeast extract, 0.75% NaCl, 0.3% NaH_2_PO_4_, and 0.75% glucose was used for *M. luteus*. Bacteria were grown overnight to the early stationary phase and adjusted in corresponding culture medium to 5.0 × 10^5^ CFU mL^−1^ in the wells of 96‐well microtiter plates, mixed with varying concentrations of test compounds, and incubated at 37 °C for 24 h. Cell growth was evaluated by measuring the optical density at 600 nm, and the MIC was defined as the lowest compound concentration at which no bacterial growth was observed.

##### Target Screening for Nisin Mutants with Activity against *E. coli*

The reported nisin analogs [nisin A, nisin Z, nisin F,^[^ ^22^ ^]^ nisin Q,^[^ ^23^ ^]^ and nisin U^[^ ^24^ ^]^] were aligned and five amino acids at non‐conserved sites (residues 4, 12, 15, 24, and 29 of nisin Z) were selected for saturation mutagenesis and inserted into pJL1 *Nde*I/*Bam*HI sites to form a mutant nisin precursor peptide library. The library was constructed with mixed plasmids, and then the mixed plasmids library was transformed into *E. coli* so that the plasmids can be separated. Randomly selected monoclonal colonies were inoculated into 250 µL per well LB with 50 µg mL^−1^ Kan in the first 96‐well plates and cultured at 37 °C for 16 h; 50 µL of *E. coli* cultures were pipetted into an equal volume of 40% sterile glycerol for preservation as a backup of each plasmid. The remaining ≈200 µL of *E. coli* cultures were centrifuged at 5000 × *g* for 20 min and the supernatant was discarded. Next, 200 µL of sterile water was added to every well for cell resuspension and 0.5 mm glass beads were added at 0.24 g per well to suspended *E. coli* cells for lysis by shaking at 220 rpm for 90 min. The first 96‐well plate was centrifuged at 4500 rpm for 5 min, 120 µL of the supernatant was pipetted into a second 96‐well plate for cryogenic centrifugal concentration (1500 rpm, –40 °C, 1 Pa), and 30 µL of sterile water was added to each well for residue resuspension. The second 96‐well plate was then centrifuged at 4500 rpm for 1 min and 4 µL of the supernatant was pipetted into 5.5 µL of the CFPS system in a third 96‐well plate, followed by incubation for 6 h at 30 °C and 220 rpm. After incubation, the CFPS mixture in the third 96‐well plate was heated at 85 °C for 5 min and centrifuged at 4500 rpm for 10 min. Then, 2 µL of the supernatant was added to the fourth 96‐well plate with 100 µL of 5.0  ×  10^5^ CFU mL^−1^ *E. coli* DH5*α* culture in each well. After adding different lanthipeptide CFPS to *E. coli*, OD_600_ was evaluated after co‐cultivation at 37 °C for 8 h.

After the first round of primary screening, those with lower OD_600_ in the same batch of experiments were considered to have the potential to inhibit the growth of *E. coli*. After the first round of primary screening, approximately five mutant strains with the lowest OD_600_ values from each 96‐well plate were selected as candidates, and ≈190 candidates among ≈3000 mutants were selected. Then, the candidate's corresponding *E. coli* backup stored in glycerol was selected for the second round of screening following the process described above. Three wells of each 96‐well plate were selected to set up the CFPS system using nisin Z plasmid as a control; to the other 93 wells in the 96‐well plate were added the candidates’ CFPS. Therefore, after 8 h of *E. coli* coincubation with the CFPS reaction system, the lowest ΔOD_600_ [ΔOD_600_ = OD_600_ (mutant) − OD_600_ (nisin Z)] were selected.

##### Fed‐Batch Fermentation of Nisin Z Production Strains

The plasmids pRL415 and pRL423 were individually transformed into the J1‐004 strain by electroporation as previously described^[^ ^39^ ^]^ to generate the engineered strains RL405 and RL406, respectively. The industrial strain, J1‐004, and colonies of engineered strains were incubated with seed medium overnight after cultivation on GM17 plates (w/v) (0.5% soy peptone, 0.5% beef extract, 0.5% tryptone, 0.25% yeast extract, 0.05% ascorbic acid, 0.025% MgSO_4_, 1.9% *β*‐glycerophosphate disodium, 0.5% d‐glucose, 1.8% agar) at 30 °C for 48 h. The seed medium (w/v) contained 1.5% peptone, 1.5% yeast extract, 1.5% sucrose, 2.0% KH_2_PO_4_, 0.15% NaCl, 0.3% corn steep liquor, 0.26% cysteine, and 0.015% MgSO_4_·7H_2_O. Then 5% of the seed culture was inoculated into 3 L of fermentation medium in a 7 L fermenter and 10 mol L^−1^ NaOH was used to adjust the medium pH to 7.2. The fermentation medium contained yeast extract (1%), sucrose (0.6%), KH_2_PO_4_ (0.5%), NaCl (0.1%), corn steep liquor (3%), cysteine (0.26%), and MgSO_4_·7 H_2_O (0.015%). The pH of the fermentation broth was maintained at 6.7 by the addition of 10 mol L^−1^ NaOH. A sucrose solution (50%, w/v) was used to maintain the residual sugar concentration in the fermentation broth at approximately 1% from 3–13 h. Samples broth (10 mL) were removed after 16 h of fermentation. After centrifugation, the supernatant was subjected to a quantitative bioassay to determine nisin production.

##### Statistical Analysis

Experiments were performed once for each individual mutant in each batch of screening nisin mutant library in CFPS platform unless otherwise indicated. Other experiments were repeated thrice independently unless otherwise indicated, statistical analysis was compiled on the means of the data obtained from at least three independent experiments. All data were expressed as the means ± standard error. “*n*” numbers for each experiment are indicated in the figure legends.

## Conflict of Interest

The authors declare no conflict of interest.

## Supporting information

Supporting Information
