# Supplementary material for: A Cell‐Free Platform Based on Nisin Biosynthesis for Discovering Novel Lanthipeptides and Guiding their Overproduction In Vivo
Source: Adv Sci (Weinh). 2020 Jul 21;7(17):2001616. doi: 10.1002/advs.202001616 (PMC7507342; doi:10.1002/advs.202001616)
Supplement: Supplementary file 1 — Supporting Information [file ADVS-7-2001616-s001.pdf]

Copyright WILEY-VCH Verlag GmbH & Co. KGaA, 69469 Weinheim, Germany, 2018.

Supporting Information

A Cell-Free Platform Based on Nisin Biosynthesis for Discovering Novel Lanthipeptides and Guiding Their Overproduction In Vivo

*Ran Liu, Yuchen Zhang, Guoqing Zhai, Shuai Fu, Yao Xia, Ben Hu, Xuan Cai, Yan Zhang, Yan Li, Zixin Deng, Tiangang Liu**

1. Supplementary Materials and Methods

Quantification of sfGFP: The fluorescence signal of sfGFP was measured according to a previously reported method[1]. In brief, measurement was performed using a microplate reader (EnSpire; PerkinElmer, Branford, CT, USA). A 40-µL volume of cell-free synthesized protein was placed in a black, flat-bottomed 96-well microplate (Greiner, Frickenhausen, Germany). Excitation and emission wavelengths were 490 and 508 nm, respectively. The absorbance value was converted to concentration (µg/mL) using standard curves generated with corresponding purified proteins.

Semi-Preparative HPLC Separation of Commercialized Nisin A: Purification was performed using the UltiMate3000 semi-preparative HPLC (Thermo Fisher). The system was equipped with a UV detector and UltiMate 3000 Fraction Collector. A Fisher Hypersil Gold column (250 × 10 mm, i.d., 5 µm) was employed for peptide separation at 35°C. The injection volume was 100 µL. The sampler tray temperature was 10°C. The column flow rate was 2 mL min^{-1}, and detection was set at 215 nm. The column was used under the following conditions: mobile phase A was H_2O (0.1% formic acid);

mobile phase B was acetonitrile (ACN). The gradient program was (time, B%) 0 min, 5% B; 1 min, 5% B; 30 min, 90% B; 35 min, 90% B; 36 min, 5% B; 50 min, 5% B. All chromatographic peaks were collected during the preliminary experiment, and all fractions were detected by LC-MS-MS. The desired factions were then collected.

LC-MS Analysis of Nisin Z and Targeted Proteomics Analysis: The original strain, J1-004, and the engineered *L. lactis* cells collected from the fermentation broth were analyzed by LC-MS for nisin Z and by a targeted proteomics approach according to a previously reported method [2]. Briefly, *L. lactis* cells collected from the fermentation broth were pelleted by centrifugation 8,000 × *g* for 10 min at 4°C, and washed thrice with wash buffer (100 mmol L^{-1} NaCl, 25 mmol L^{-1} Tris−HCl, pH 7.5). The wet cell pellet was suspended in an equal volume (1 g wet cell weight/1 mL of buffer) of lysis buffer comprised of 8 mol L^{-1} urea, 2 mol L^{-1} thiourea, 75 mmol L^{-1} NaCl, 4% (w/v) 3-[(3-cholamidopropyl)-dimethylammonio]-1-propanesulfonate (CHAPS), 50 mmol L^{-1} Tris−HCl (pH 8.0) and one complete EDTA-free protease inhibitor cocktail tablet (Roche, Indianapolis, IN, USA) per 10 mL of buffer. The suspended sample was vortexed for 30 s twice and disrupted by sonication [3]. The supernatant from the lysed cells was collected by centrifugation (13,000 × *g* for 45 min at 4°C). Proteins from the cell lysates were measured using a noninterference protein assay kit (Sangon Biotech, Shanghai, China) and adjusted to 2 μg/μL using lysis buffer. First, 50 μL of the supernatant (100 μg of total protein) was mixed with an equal volume of 100 mM ammonium bicarbonate buffer (pH 8.0). Next, the sample was reduced at 30°C for 1 h by the addition of 3 mmol L^{-1} tris (2-carboxyethyl)-phosphine (TCEP) and alkylated by the addition of 15 mmol L^{-1} iodoacetamide (IAA). The samples were incubated in dark conditions at 30°C for an additional 1 h. The sample was diluted with ammonium bicarbonate buffer to reduce the urea concentration to 1 mol L^{-1}. Trypsin was added to the mixture (trypsin/total protein 1:50, w/w) and incubated at 37°C for 14 h. The detergent and salt in the digested peptide sample were removed by passage through a Pierce Detergent Removal Spin Column (Thermo Fisher Scientific) and a SepPak C18 cartridge (Waters Corp.), respectively.

The purified peptides were freeze-dried and stored at -80°C for subsequent liquid chromatography-tandem mass spectrometry (LC-MS-MS) analysis.

The peptide samples were analyzed using a hybrid quadrupole-time-of-flight (TOF) liquid chromatography (LC) tandem mass (MS/MS) spectrometer (TripleTOF 5600+, AB Sciex, Foster City, CA, USA) equipped with a nanospray ion source. Peptides were first loaded onto a C18 trap column (5 μm, 5 × 0.3 mm; Agilent Technologies, Santa Clara, CA, USA) and then eluted into a C18 analytical column (75 μm × 150 mm, 3 μm particle size, 100 Å pore size; Eksigent, Dublin, CA, USA). Mobile phase A (3% DMSO, 97% H_2O, 0.1% formic acid) and mobile phase B (3% DMSO, 97% ACN, 0.1% formic acid) were used to establish a 100-min gradient as follows: 0 min of 5% B, 65 min of 5–23% B, 20 min of 23–52% B, 1 min of 52–80% B, maintenance at 80% B for 4 min, 0.1 min of 80–5% B, and a final step of 5% B for 10 min. A constant flow rate was set at 300 nL/min. MS scans were conducted from 350 amu-1500 amu, with a 250-ms time span. For the MS/MS analysis, each scan cycle consisted of one full-scan mass spectrum (with m/z ranging from 350-1500 and charge states from 2-5) followed by 40 MS/MS events. The threshold count was set to 120 to activate MS/MS accumulation and former target ion exclusion was set to 18 s. Raw data obtained by TripleTOF 5600+ were analyzed using ProteinPilot 5.0 (AB SCIEX) against the designated proteome database.

2. Supplementary Figures

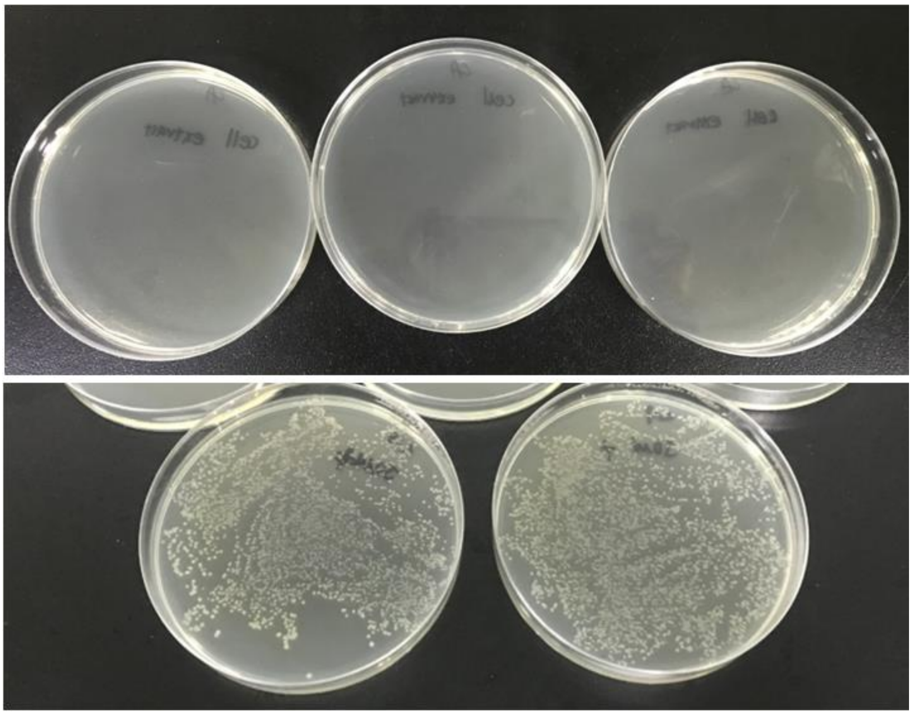

Figure S1. Cytoplasmic Extract Control Plates All LB agar (no antibiotic) plates were incubated at 37°C for 16 h and 27 % v/v of the cell extract was used for each CFPS reaction. **Top:** Approximately 50 μL of *E. coli* cell extracts was plated on each of the three plates. No *E. coli* colonies grew on either plate. **Bottom:** Approximately 50 μL of *E. coli* (5.0×10^5 CFU mL^{-1}) cells were added to the plate as a positive control.

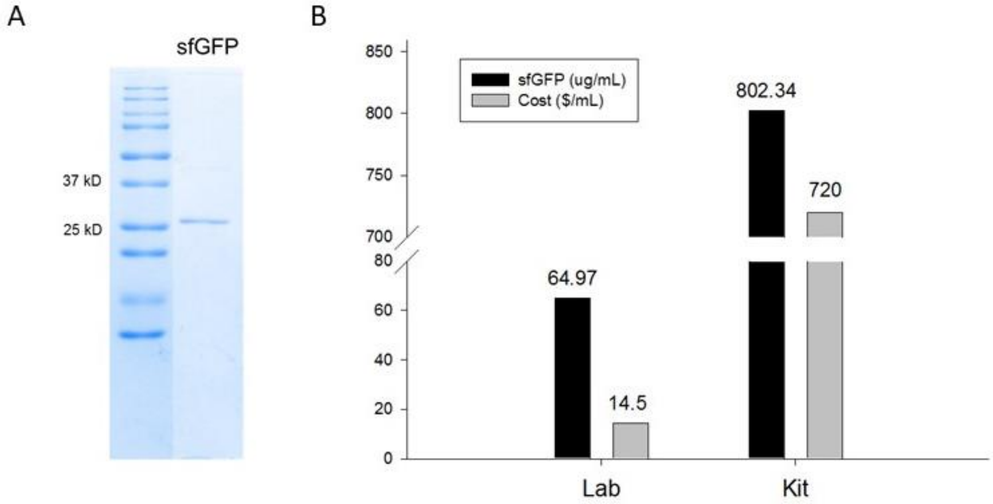

Figure S2. Comparison of the performance of our cell-free system with a commercial cell-free synthesis kit. (A) Purified sfGFP was used as the standard, which was purified according to our previously reported method. [1] (B) The titer of sfGFP and the cost of cell-free reagents in our cell-free system and the commercial cell-free synthesis kit (RTS 100 *E. coli* Disulfide Kit; Biotechrabbit, Berlin, Germany). 100 ng of pJL1-*sfGFP* was added to 40 μL of the cell-free system and the commercial cell-free synthesis kit, respectively. The cell-free reaction was conducted according to our described method or kit instructions, respectively. The calculation of cost was based on of price for each raw material or kit in this study. After incubation for 6 h, the titer of sfGFP produced by our cell-free system was 64.97 mg L^{-1}, and that produced by the commercial kit was 802.34 mg L^{-1}. The commercial cell-free synthesis kit has been demonstrated to result in very high productivity; therefore, the 12-fold lower titer of sfGFP in our system indicated the acceptable efficiency of our system. Moreover, since our cell-free system is about 50-fold cheaper than the commercial kit, a scale-up study is feasible. Overall, this comparison demonstrated that our system can meet the needs of subsequent research.

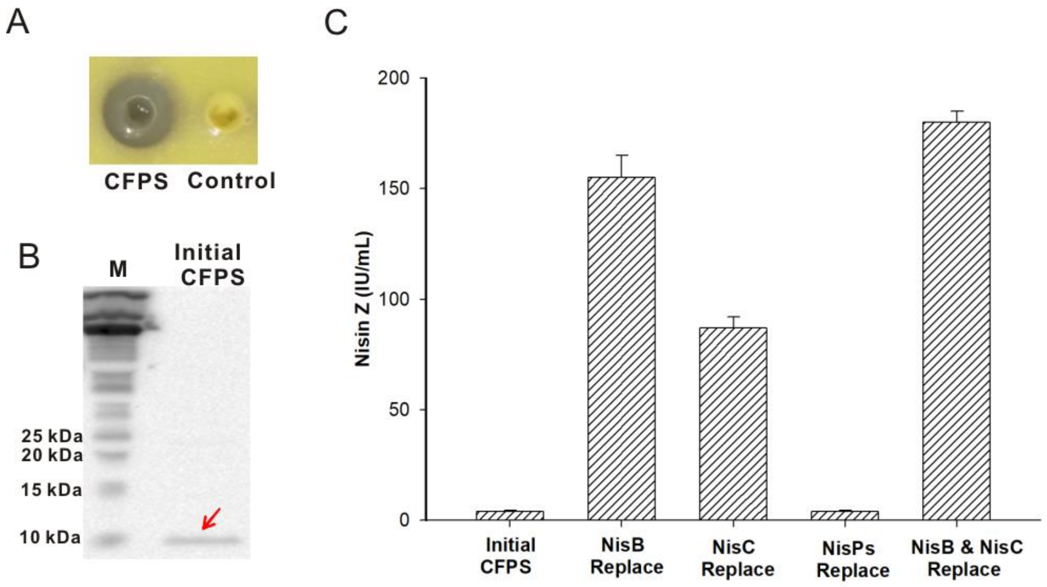

Figure S3. Analysis of the Nisin CFPS System (A) Validation of antimicrobial activity by the agar diffusion assay. An obvious zone of inhibition was detected on the *M. luteus* plate using the nisin CFPS reaction mixture (CFPS). Control: CFPS reaction without pJL1-*nisZ*, pET28a-*nisB*, pET28a-*nisC*, and pET28a-*nisP*. (**B**) Western blot analysis of expressed proteins (NisZ, NisB, NisC, and NisP) involved in Nisin CFPS. Red arrow indicates the partially modified nisin precursor with an N-terminal His6-tag. A primary anti-His6 tag mouse monoclonal antibody was diluted 1:2,000 in blocking buffer (2% nonfat milk in TBST) before use. Horseradish peroxidase (HRP)-conjugated goat anti-mouse IgG was used as the secondary antibody, and the SuperSignal West Pico Plus chemiluminescent substrate was used to visualize proteins via chemiluminescence. (**C**) Replacement of poorly expressed protein encoding plasmids with purified enzymes in CFPS (n=3). The final concentrations of purified proteins were fixed at 500 nM. The data represented mean ± SD. 40 IU=1 μg nisin.

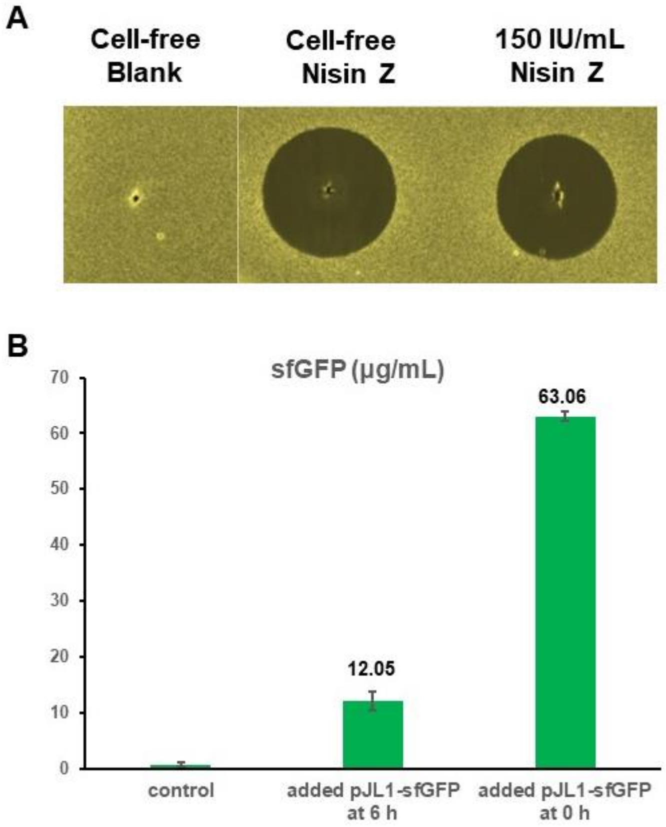

Figure S4. Production of nisin or sfGFP in different cell-free systems. (A) Validation of antimicrobial activity by the agar diffusion assay. Cell-free blank: CFPS reaction without pJL1-*nisZ*, pET28a-*nisB*, pET28a-*nisC*, and pET28a-*nisP*. Cell-free nisin Z: pJL1-*nisZ* (0.13 nmol L^{-1}), NisB (800 nmol L^{-1}), NisC (500 nmol L^{-1}), and pET28-*nisP* (0.1 nmol L^{-1}) were added to the cell-free system. After 6 h of incubation, 2 μL of the reaction mixture was used in a bioassay with *M. luteus* as the indicator strain. (B) Production of sfGFP in different cell-free systems (n=3). Control: blank cell-free system incubated for 6 h; added pJL1-*sfGFP* at 6 h: followed by the addition of pJL1-*nisZ* (0.13 nmol L^{-1}), NisB (800 nmol L^{-1}), NisC (500 nmol L^{-1}), and pET28a-*nisP* (0.1 nmol L^{-1}) and incubation for 6 h, and then 100 ng pJL1-*sfGFP* was added to the system and incubated for another 6 h. added pJL1-*sfGFP* at 0 h: 100 ng of pJL1-*sfGFP* was added into cell-free system at 0 h and incubated for 6 h. The data represented mean ± SD.

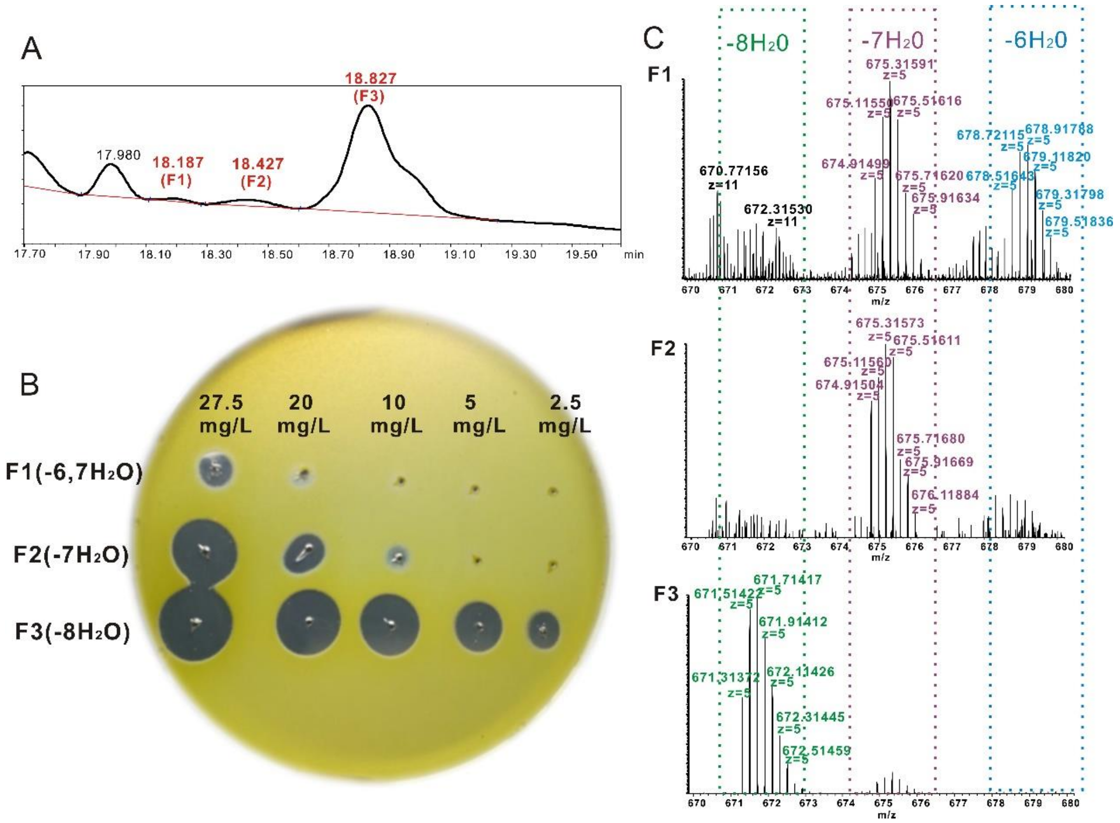

Figure S5. Identification of the Most Efficient Nisin A Component (**A**) Separation of different components of nisin A standard by semi-preparative HPLC. F1, F2, and F3 were collected. (**B**) Antibacterial activity assay of different components using the agar diffusion method. The concentrations of F1, F2, and F3 were set to 2.5 mg/L, 5 mg/L, 10 mg/L, 20 mg/L, and 27.5 mg/L, and 2 μL of each sample was used for the antibacterial activity test. (**C**) High-resolution mass spectrometry of different components. The F1 component consists of dehydrated (-6, -7H_2O) nisin A, the F2 component consists of dehydrated (-7 H_2O) nisin A, and the F3 component consists of dehydrated (-8 H_2O) nisin A.

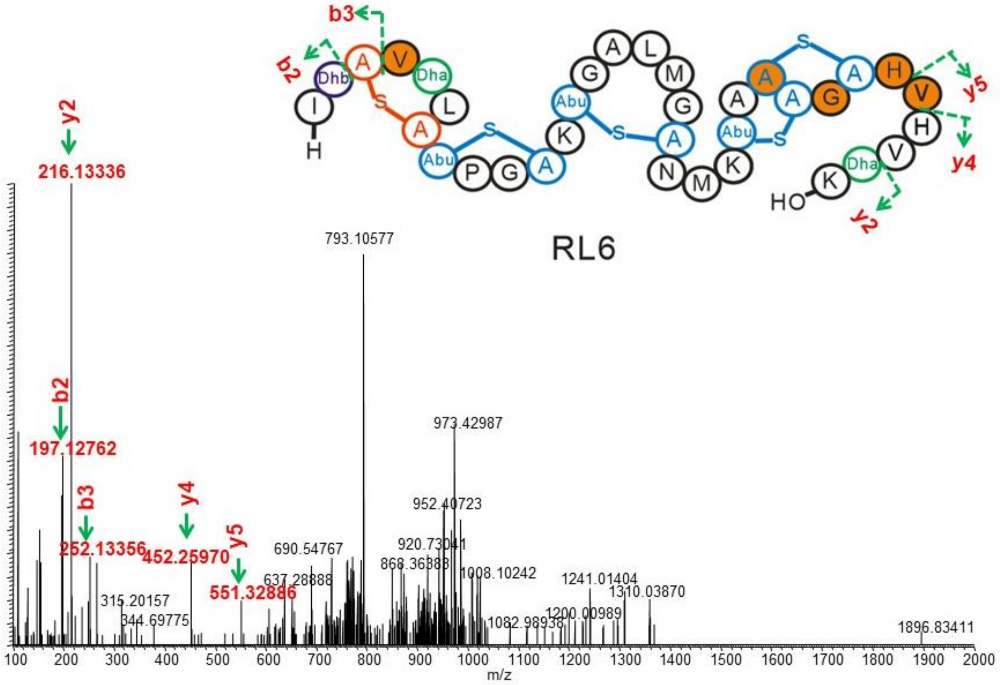

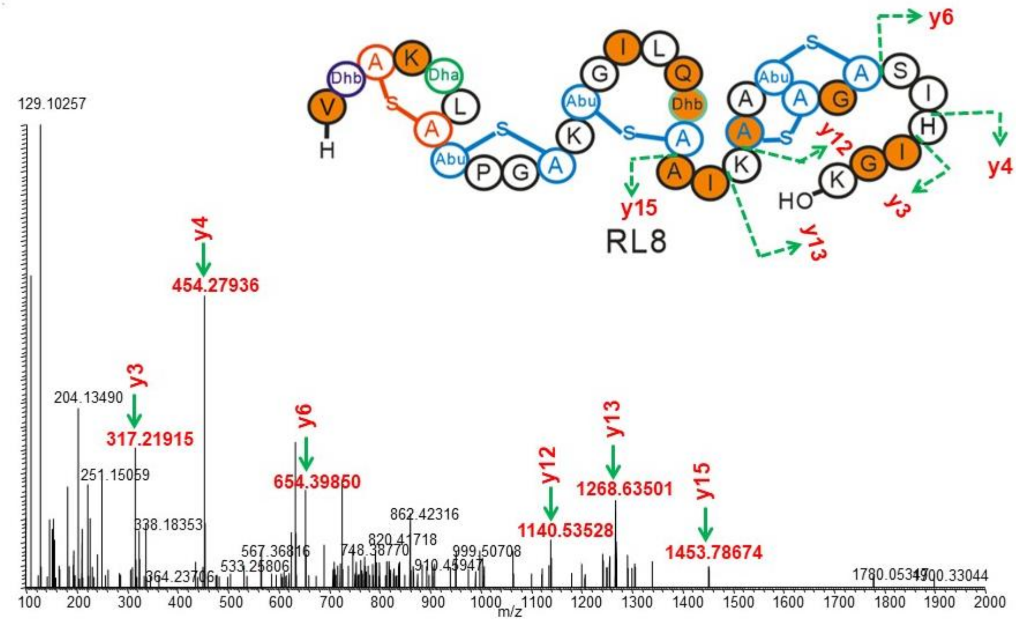

Figure S6. Continued on next page.

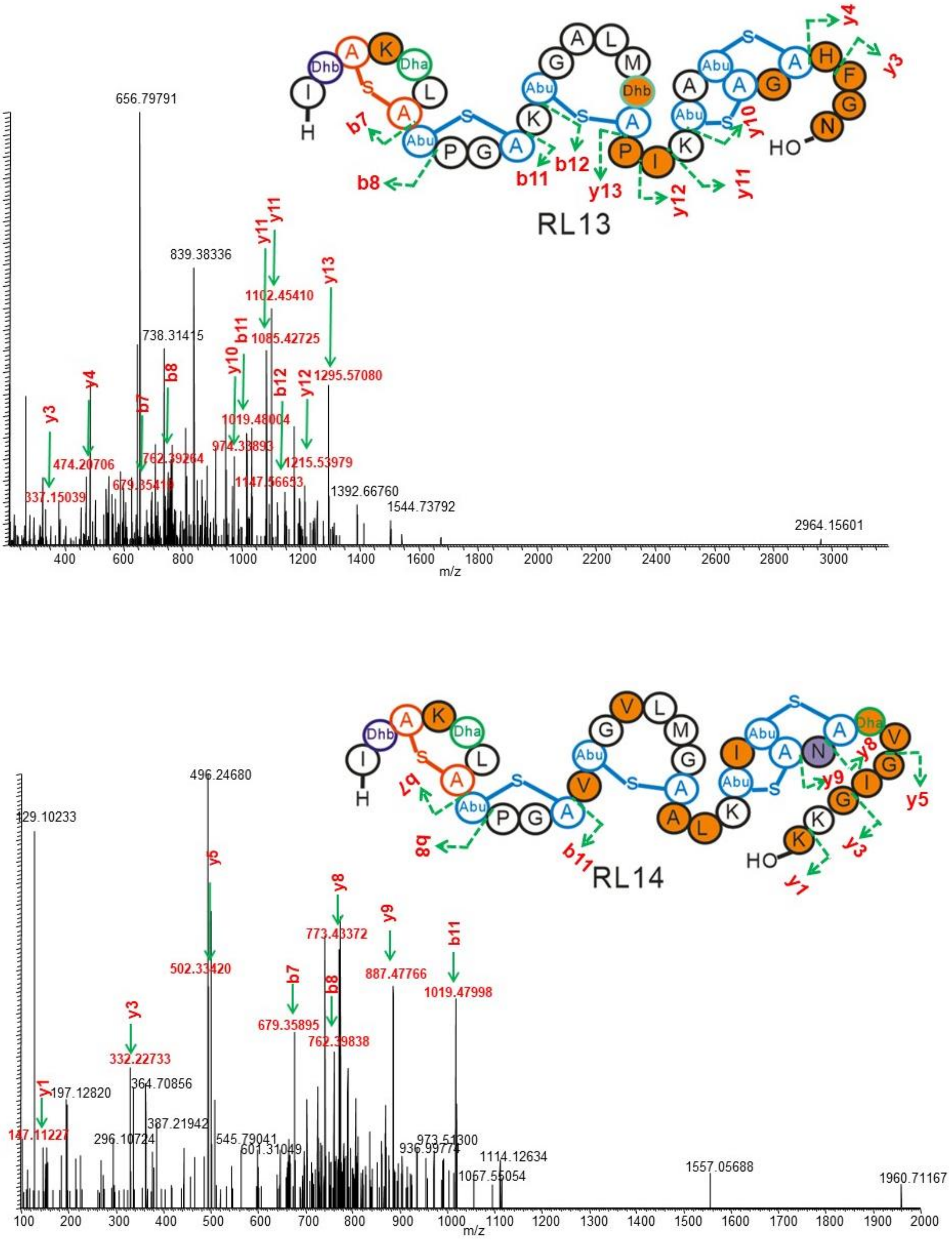

Figure S6. Continued on next page.

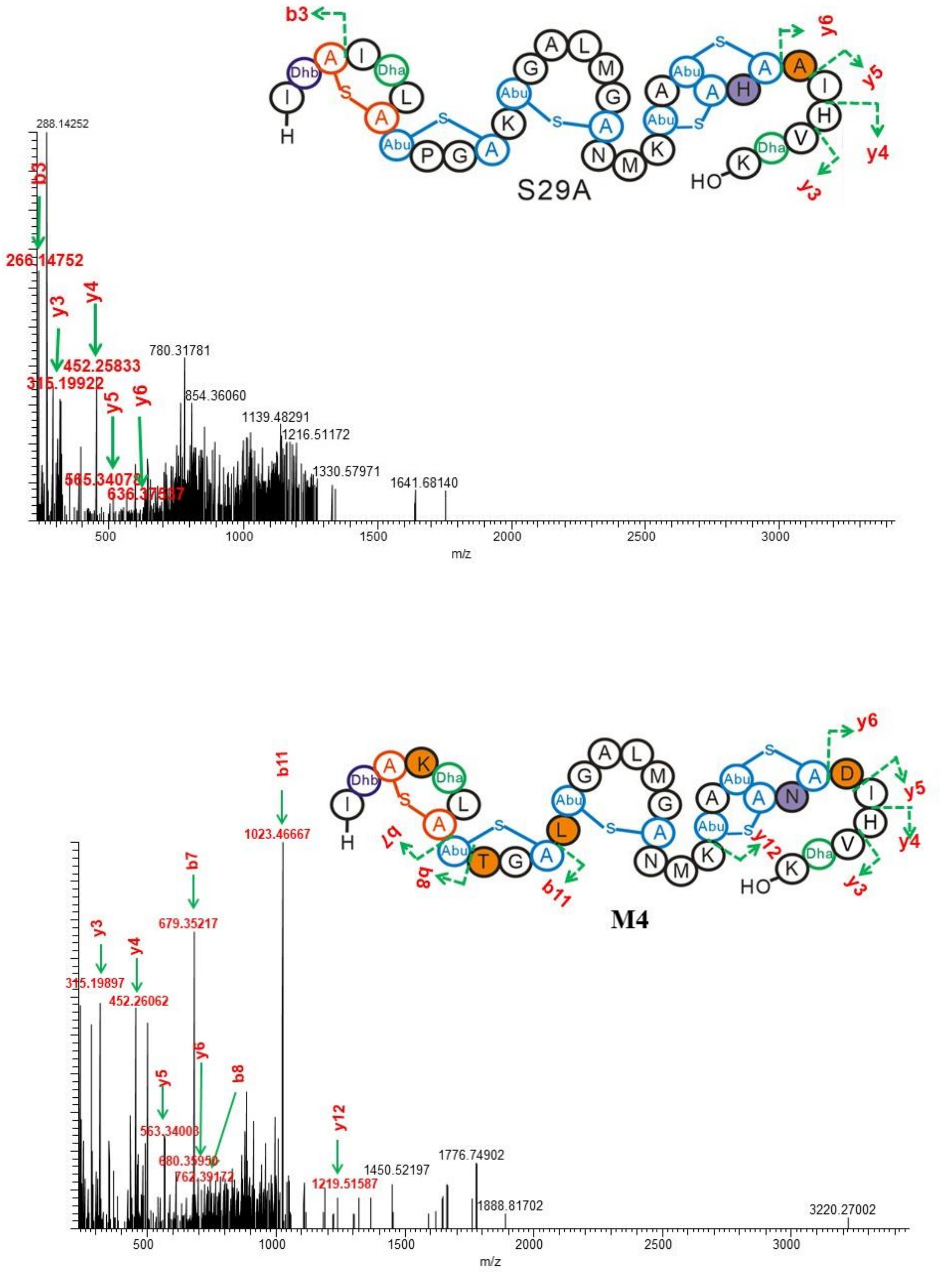

Figure S6. Continued on next page.

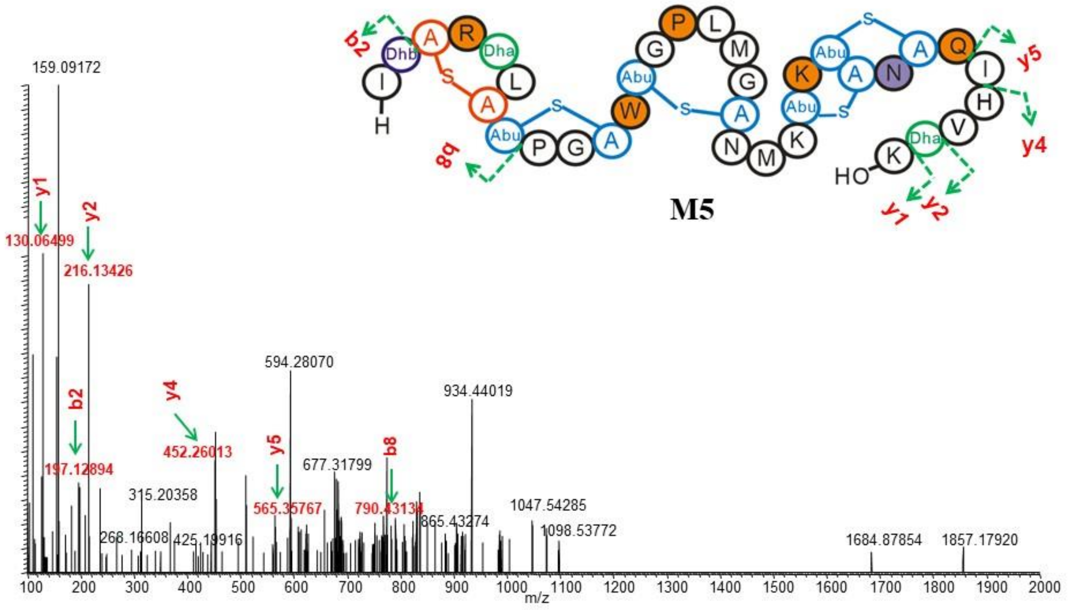

Figure S6. Structure of Nisin Analogs and Nisin Mutants Determined by High-Resolution Mass Spectrometry Brown circle markers represent the amino acid residues that are different from nisin residues. Dha, dehydrolalanine; Dhb, dehydrobutyrine; Abu, 2-aminobutyric acid.

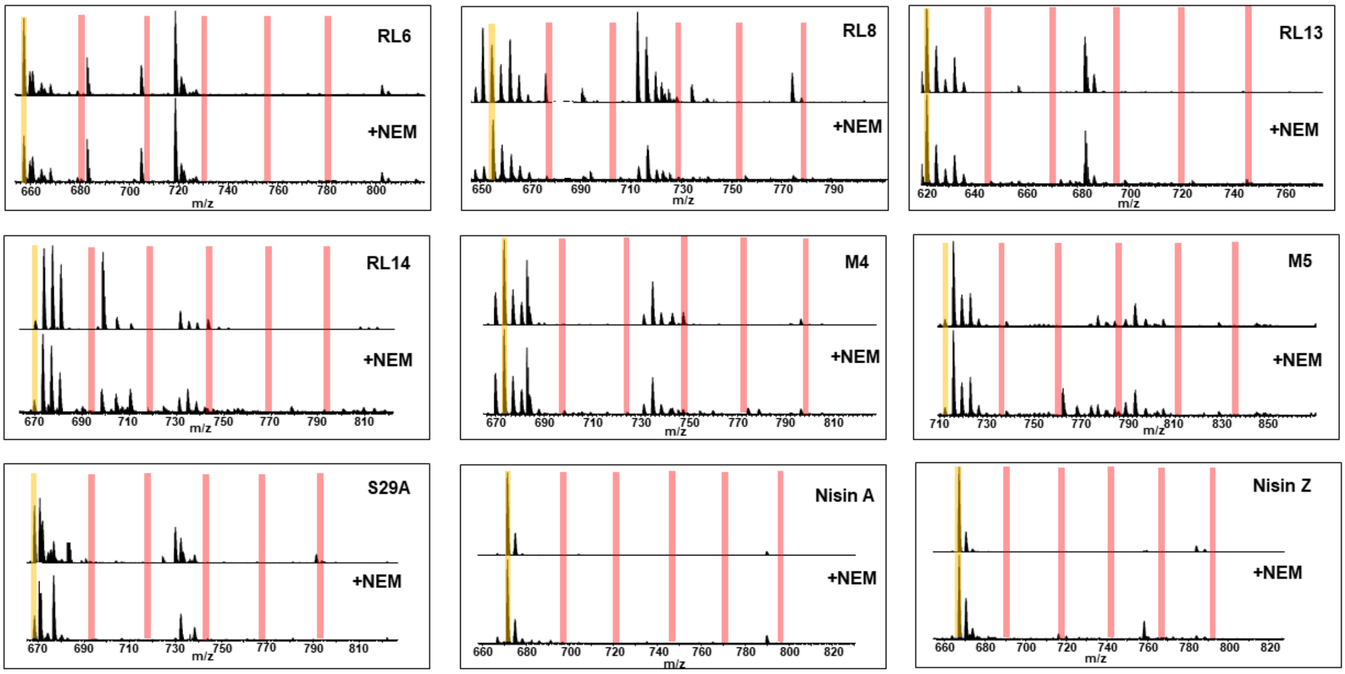

Figure S7. Summary of LC-MS Analysis of Lanthipeptides All ions are in the +5-charge state. Purified modified precursor peptides were digested with trypsin to form mature RL6, RL8, RL13, RL14, M4, M5, and S29A and treated with NEM. Commercialized nisin A and nisin Z were also treated with NEM. The thioether crosslinks are formed via a Michael-type addition by reducing cysteine thiols (-SH) in Cys residues to dehydro-amino acids. This step does not cause any change in molecular weight between substrates and products. However, if there is a Cys in the protein that does not form a thioether crosslink, there will be a complete -SH on the peptide. If the -SH undergoes an alkylation reaction with NEM, the product will have a larger molecular mass (125 Da) than the substrate. The eight-fold dehydrated core peptides are highlighted in yellow. Theoretical alkylated core peptides with 1–5 NEM adducts are highlighted in red. The results showed all five thioester rings were formed in mature lanthipeptides.

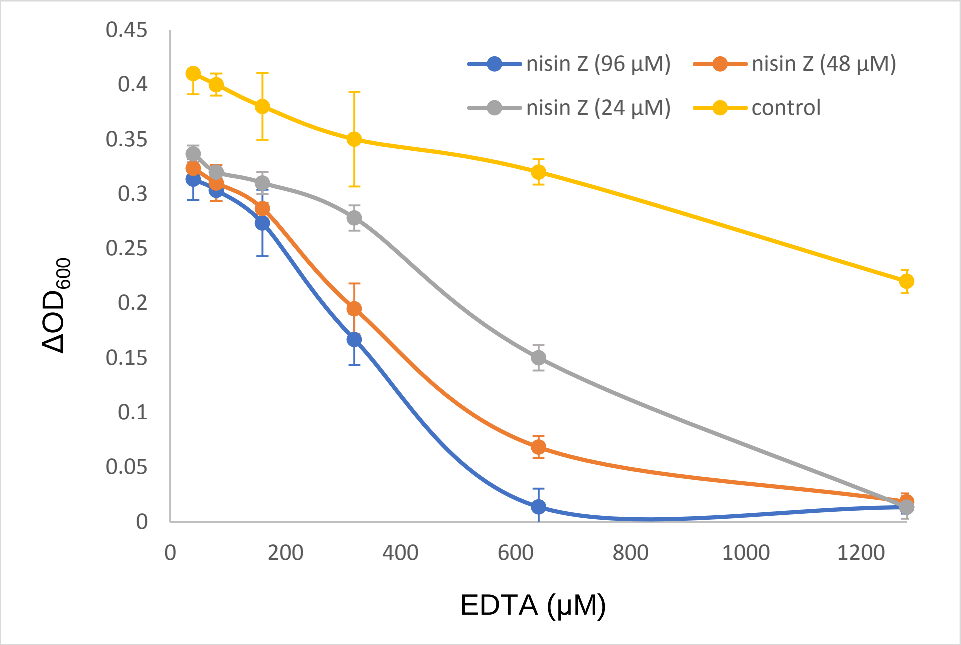

Figure S8. OD_{600} of *E. coli* DH5α in LB Medium with Different Concentrations of EDTA and Nisin after 18 h of Co-culture (n=3) ΔOD_{600} indicates the difference between readings with different concentrations of blank media. Previous studies have reported that nisin has a significant inhibitory effect on *E. coli* [4], however, uncommon *E. coli* strains were used and they are available in our laboratory. In the reported 20-fold nisin concentration condition, DH5α did not exhibit complete inhibition. To enable comparisons with previous studies, EDTA was added to the *E. coli* culture to increase the sensitivity of *E. coli* to nisin. After 18 h, *E. coli* DH5α growth was only slightly inhibited following addition of 320 μM EDTA, however, the inhibitory effects of various concentrations of nisin on *E. coli* were obvious. The data represented mean ± SD.

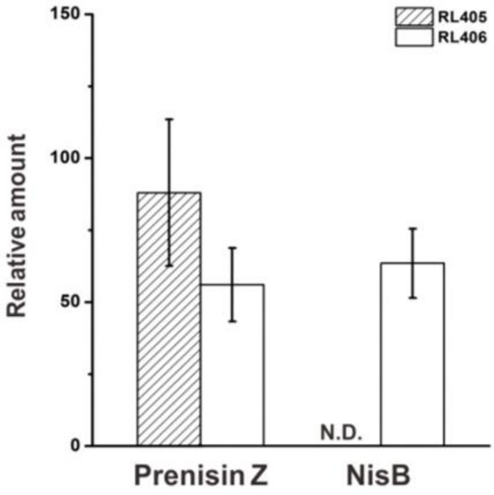

Figure S9. Proteomic Analysis of Targeted Proteins Involved in Nisin Biosynthesis in Engineered Strains (n=3) Relative amount: number of identified specific peptides with 95% confidence; N.D., not detected. Error bars are based on three independent replicates. The data represented mean ± SD.

2. Supplementary Tables

Table S1. Gene Sequences of Hybrid Precursor Peptides

>RL1

ATGAGTACAAAAGATTTTAACTTGGATTTGGTATCTGTTTCGAAGAAAGATTCAGGTG

CATCACCACGCATCACCGTGCGTAGCAAGAGCCTGTGCACCCCGGGTTGCATTACCGG

TCCGCTGCGTACCTGCTACCTGTGCTTCCCGACCCACGTTAACTGCTAA

>RL2

ATGAGTACAAAAGATTTTAACTTGGATTTGGTATCTGTTTCGAAGAAAGATTCAGGTG

CATCACCACGCATCACCTGGAAGAGCGAGAGCCTGTGCACCCCGGGTTGCGTGACCGG

CGTTCTGCAGACCTGCTTCCTGCAAACCATCACCTGCAACTGCAAGATTAGCAAATAA

>RL3

ATGAGTACAAAAGATTTTAACTTGGATTTGGTATCTGTTTCGAAGAAAGATTCAGGTG

CATCACCACGCGTGACCAGCAAGAGCCTGTGCACCCCGGGTTGCATCACCGGCATTCT

GATGTGCCTGACCCAGAACAGCTGCGTTAGCTGCAACAGCTGCATCAAATGCTAA

>RL4

ATGAGTACAAAAGATTTTAACTTGGATTTGGTATCTGTTTCGAAGAAAGATTCAGGTG

CATCACCACGCATCACCAGCAAAAGCCTGTGCACCCCGGGTTGCGTGACCGGCATTCT

GATGACCTGCCCGGTTCAGACCGCGACCTGCGGTTGCCAAATCACCGGCAAATAA

>RL5

ATGAGTACAAAAGATTTTAACTTGGATTTGGTATCTGTTTCGAAGAAAGATTCAGGTG

CATCACCACGCATCACCAGCAAGAGCCTGTGCACCCCGGGTTGCATCACCGGCATTCT

GATGTGCCTGACCCAGAACAGCTGCGTGAGCTGCAACAGCTGCATTCGTTGCTAA

>RL6

ATGAGTACAAAAGATTTTAACTTGGATTTGGTATCTGTTTCGAAGAAAGATTCAGGTG

CATCACCACGCATCACCAGCGTGAGCCTGTGCACCCCGGGTTGCAAGACCGGTGCGCT

GATGGGTTGCAACATGAAAACCGCGAGCTGCGGCTGCCACGTGCACGTTAGCAAGTAA

>RL7

ATGAGTACAAAAGATTTTAACTTGGATTTGGTATCTGTTTCGAAGAAAGATTCAGGTG

CATCACCACGCATCACCAGCGTGAGCCTGTGCACCCCGGGTTGCGTGACCGGCGTTCT

GATGTGCCCGGGTAACACCATTAGCTGCAACGGCCACTGCAGCATCCACATTACCGGT

TAA

>RL8

ATGAGTACAAAAGATTTTAACTTGGATTTGGTATCTGTTTCGAAGAAAGATTCAGGTG

CATCACCACGCGTGACCAGCAAGAGCCTGTGCACCCCGGGTTGCAAAACCGGCATCCT

GCAGACCTGCGCGATTAAGAGCGCGACCTGCGGTTGCAGCATCCACATTGGCAAATAA

>RL9

ATGAGTACAAAAGATTTTAACTTGGATTTGGTATCTGTTTCGAAGAAAGATTCAGGTG

CATCACCACGCGTGACCAGCAAGAGCCTGTGCACCCCGGGTTGCATCACCGGCGTGCT

GATGTGCCTGACCCAGAACAGCTGCGTTAGCTGCAACAGCTGCATTAAATGCTAA

>RL10

ATGAGTACAAAAGATTTTAACTTGGATTTGGTATCTGTTTCGAAGAAAGATTCAGGTG

CATCACCACGCATCACCGTGAAGATTACCAGCTACAGCCTGTGCACCCCGGGTTGCAA

GACCGGTGCGCTGATGGGTTGCACCATGAAAACCGCGAGCTGCGGCTGCCACGTTCAC

ATCAGCAAATAA

>RL11

ATGAGTACAAAAGATTTTAACTTGGATTTGGTATCTGTTTCGAAGAAAGATTCAGGTG

CATCACCACGCATCACCTGGAAGAGCGAGAGCCTGTGCACCCCGGGTTGCATTACCGG

CGTGCTGCAGACCTGCTTCCTGCAAACCATCACCTGCAACTGCCACATTAGCAAATAA

>RL12

ATGAGTACAAAAGATTTTAACTTGGATTTGGTATCTGTTTCGAAGAAAGATTCAGGTG

CATCACCACGCATCACCAGCTACAGCCTGTGCACCCCGGGTTGCATTACCGGCGTTCTG

ATGGGTTGCCACATCCAGAGCATTGGCTGCAACGTGCACGTTCACGTGAGCAAGTAA

>RL13

ATGAGTACAAAAGATTTTAACTTGGATTTGGTATCTGTTTCGAAGAAAGATTCAGGTG
CATCACCACGCATCACCAGCAAGAGCCTGTGCACCCCGGGTTGCAAAACCGGCGCGCT
GATGACCTGCCCGATTAAGACCGCGACCTGCGGTTGCCACTTCGGCAACTAA

>RL14

ATGAGTACAAAAGATTTTAACTTGGATTTGGTATCTGTTTCGAAGAAAGATTCAGGTG
CATCACCACGCATCACCAGCAAGAGCCTGTGCACCCCGGGTTGCGTGACCGGCGTTCT
GATGGGTTGCGCGCTGAAAACCATCACCTGCAACTGCAGCGTGGGTATTGGCAAGAAA
TAA

>RL15

ATGAGTACAAAAGATTTTAACTTGGATTTGGTATCTGTTTCGAAGAAAGATTCAGGTG
CATCACCACGCATCACCAGCAAGAGCCTGTGCACCCCGGGTTGCGTTACCGGTCTGCT
GATGGGTTGCGCGGGTAGCAGCGCGACCTGCAACTGCAGCGTGCACGTTGGTTAA

>RL16

ATGAGTACAAAAGATTTTAACTTGGATTTGGTATCTGTTTCGAAGAAAGATTCAGGTG
CATCACCACGCATCACCAGCAAGAGCCTGTGCACCCCGGGTTGCGTGACCGGCGTTCT
GATGGGTTGCAACAACAAAACCGCGACCTGCAACTGCAGCGTGCACGTTGGCTAA

>RL17

ATGAGTACAAAAGATTTTAACTTGGATTTGGTATCTGTTTCGAAGAAAGATTCAGGTG
CATCACCACGCATCACCCAGTTCAAGAGCATTAGCCTGTGCACCCCGGGTTGCCCGAC
CGGTATCCTGATGGGTTGCCATAAGTGCCCGAGCGGTAGCGACACCGTGTACACCAAA
TAA

>RL18

ATGAGTACAAAAGATTTTAACTTGGATTTGGTATCTGTTTCGAAGAAAGATTCAGGTG
CATCACCACGCATCACCAGCCCGCAGATTACCAGCGTGAGCCTGTGCACCCCGGGTTG

CCAGACCGGCTTCCTGGCGTGCTTTAGCCAAGCGTGCAACCCGACCGGTGGCTGCAAG

ATCAGCAAATAA

>NisZ

ATGAGTACAAAAGATTTTAACTTGGATTTGGTATCTGTTTCGAAGAAAGATTCAGGTG

CATCACCACGCATCACCAGTATTTCGCTATGTACACCCGGTTGTAAAACAGGAGCTCT

GATGGGTTGTAACATGAAAACAGCAACTTGTAATTGTAGTATTCACGTAAGCAAATAA

> Bagelicin

ATGAGTACAAAAGATTTTAACTTGGATTTGGTATCTGTTTCGAAGAAAGATTCAGGTG

CATCACCACGCGTGACCAGCATCAGCCTGTGCACCCCGGGTTGCAAGACCGGCATCCT

GATGACCTGCGCGATTAAAACCGCGACCTGCGGTTGCCACTTCTAA

>M4

ATGAGTACAAAAGATTTTAACTTGGATTTGGTATCTGTTTCGAAGAAAGATTCAGGTG

CATCACCACGCATTACAAGTAAGTCGCTATGTACAACCGGTTGTCTGACAGGAGCGCT

GATGGGTTGTAACATGAAAACAGCGACTTGTAATTGTGATATTCACGTAAGCAAATAA

>M5

ATGAGTACAAAAGATTTTAACTTGGATTTGGTATCTGTTTCGAAGAAAGATTCAGGTG

CATCACCACGCATTACAAGTCGGTCGCTATGTACACCCGGTTGTTGGACAGGACCTCT

GATGGGTTGTAACATGAAAACAAAGACTTGTAATTGTCAGATTCACGTAAGCAAATAA

>S29A

ATGAGTACAAAAGATTTTAACTTGGATTTGGTATCTGTTTCGAAGAAAGATTCAGGTG

CATCACCACGCATCACCAGCATTAGCCTGTGCACCCCGGGTTGCAAGACCGGTGCGCT

GATGGGTTGCAACATGAAAACCGCGACCTGCCACTGCGCGATCCACGTGAGCAAGTAA

Note: leader peptide sequence is gray marked, core peptide sequence is underlined.

Table S2. Bacterial Strains

Strains	Describe	Source
DH10β	Plasmid construction	Our Lab Preserve
XL1-Blue	Plasmid construction	Our Lab Preserve
BL21(DE3)	Protein/ mLanAs overexpression.	Our Lab Preserve
BL21Rosseta(DE3)	NisPs overexpression.	Our Lab Preserve
Micrococcus luteus	Indicator strain for nisin antibacterial activity test	NCIB 8166
BL21(DE3)/pYZ95/pYX126	Expression of sumo-tagged mRL6	This study
BL21(DE3)/pYZ96/pYX126	Expression of sumo-tagged mRL8	This study
BL21(DE3)/pYX122/pYX125	Expression of sumo-tagged mRL13	This study
BL21(DE3)/pYX123/pYX125	Expression of sumo-tagged mRL14	This study
BL21(DE3)/pYZ92/pYX126	Expression of sumo-tagged mM4	This study
BL21(DE3)/pYZ93/pYX126	Expression of sumo-tagged mM5	This study
BL21(DE3)/pYZ97/pYX126	Expression of sumo-tagged S29A	This study
Enterococcus faecalis	Clinical standard strain for antibacterial testing	ATCC 29212
Staphylococcus aureus	Clinical standard strain for antibacterial testing	ATCC 25923
Methicillin-resistant *Staphylococcus aureus* (MRSA)	Clinical isolation of antibiotic test resistant strains	Renmin hospital of Wuhan University
Lactococcus lactis J1-004	Nisin Z industrial producing strain	J1 Biotech. Co.
RL405	J1-004/pRL415, Overexpress *nisZ* in J1-004	This study
RL406	J1-004/pRL423, Overexpress nisZ and *nisB* in J1-004	This study
BL21(DE3)/ pYX106	Expression of His6-tagged mRL6 (with one copy of *nisB* overexpressed) in *E. coli*	This study

BL21(DE3)/ pYX106//pYX125	overexpress His6-tagged mRL6 (with two copies of *nisB* overexpressed) in *E. coli*	This study

Table S3. MIC Values of Several Lanthipeptides Against Microorganisms

MIC* mg/L(μM)	*M. luteus*	*E. faecalis*	*S. aureus*	MRSA
Nisin A	0.06 (0.018)	250 (74.53)	125 (37.27)	125 (37.27)
Nisin Z	0.12 (0.036)	250 (75.05)	250 (75.05)	125 (37.52)
RL6	0.12 (0.037)	250 (76.17)	>250 (>76.17)	>250 (>76.17)
RL8	0.24 (0.073)	250 (76.52)	>250 (>76.52)	>250 (>76.52)
RL13	0.24 (0.077)	250 (80.8)	>250 (>80.8)	>250 (>80.8)
RL14	0.015 (0.0045)	62.5 (18.69)	250 (74.75)	250 (74.75)

Note: * The test lanthipeptide is a mixture of different dehydration molecules. Quantification of lanthipeptides was performed using the eight-fold dehydrated molecules (n=3). The data represented mean ± SD.

Table S4. Primers Used for Construction Plasmids

Primer	Primers for PCR
**Primers of plasmids for CFPS biosynthesis**	
pJL1-*nisZ*-F	5'-GGGTTTCATATGAGTACAAAAGATTTTAACTTG-3'
pJL1-*nisZ*-R	5'- AATTGGATCCTTATTTGCTTACGTGAATACTAC -3'
pET28a-*nisB*-F	5'-GGGTTTCATATGATAAAAAGTTCATTTAAAGC-3'
pET28a-*nisB*-R	5'-GCGCGGATCCTCATTTCATGTATTCTTCCGAAAC-3'
pET28a-*nisC*-F	5'-GCGCGGATCCATGAATAAAAAAAATATAAAAAG-3'
pET28a-*nisC*-R	5'-ATATAAGCTTTCATTTCCTCTTCCCTCCTTTC-3'
pET28a-*nisP*-F	5'-GGGTTTCATATGAAAAAAATACTAGGTTTCC-3'
pET28a-*nisP*-R	5'-ATATGGATCCTCAATTTTTAGTCTTTCTTTTC-3'
pET28a-*nisPs*-F	5'-AGCACCATGGATGAAAAAAATACTAGGTTTCCT-3'
pET28a-*nisPs*-R	5'-AGCACTCGAGTTAGTGGTGGTGGTGGTGGTGGTGTTGACTTCGTACAGAAACAGCA-3'
pRL-F	5'-GCAGCCATATGAGTACAAAAGATTTTAAC-3'
pRL1-R	5'- ATTCGGATCCTTAGCAGTTAACGTGGGTCG-3'
pRL2-R	5'- ATTCGGATCCTTATTTGCTAATCTTGCAGTTG-3'
pRL3-R	5'- ATTCGGATCCTTAGCATTTGATGCAGCTGTTG-3'
pRL4-R	5'- ATTCGGATCCTTATTTGCCGGTGATTTGGCA -3'
pRL5-R	5'- ATTCGGATCCTTAGCAACGAATGCAGCTGTTG-3'
pRL6-R	5'- ATTCGGATCCTTACTTGCTAACGTGCACGTG-3'
pRL7-R	5'- ATTCGGATCCTTAACCGGTAATGTGGATGCTG-3'
pRL8-R	5'- ATTCGGATCCTTATTTGCCAATGTGGATGCTG -3'
pRL9-R	5'- ATTCGGATCCTTAGCATTTAATGCAGCTGTTG -3'
pRL10-R	5'- ATTCGGATCCTTATTTGCTGATGTGAACGTG-3'
pRL11-R	5'- ATTCGGATCCTTATTTGCTAATGTGGCAGTTG-3'
pRL12-R	5'- ATTCGGATCCTTACTTGCTCACGTGAACGTG-3'
pRL13-R	5'- ATTCGGATCCTTAGTTGCCGAAGTGGCAAC-3'
pRL14-R	5'- ATTCGGATCCTTATTTCTTGCCAATACCCAC-3'

pRL15-R	5'- ATTCGGATCCTTAACCAACGTGCACGCTGCA-3'
pRL16-R	5'- ATTCGGATCCTTAGCCAACGTGCACGCTGCA-3'
pRL17-R	5'- ATTCGGATCCTTATTTGGTGTACACGGTGTC-3'
pRL18-R	5'- ATTCGGATCCTTATTTGCTGATCTTGCAGCCA-3'
pJL1-bagelicin-R	5'- ATTCGGATCCTTAGAAGTGGCAACCGCAGGT-3'

Primers of plasmids for mLanA overexpression in *E. coli*

pYZ82-F	5'-GCAACATATGATAAAAAGTTCATTTAAAGCTCAA-3'
pYZ82-R	5'-AGCAGGTACCTCATTTCATGTATTCTTCCGAAAC -3'
pYZ(85-89)-F	5'-AGCAGGATCCAATGAGTACAAAAGATTTTAACTTG-3'
pYZ85-R	5'-CGATGAATTCTTACTTGCTAACGTGCACGT-3'
pYZ86-R	5'-CGATGAATTCTTATTTGCCAATGTGGATGC-3'
pYZ87-R	5'-AGCAGAATTCTTACTTGCTCACGTGGATCGCGCA-3'
pYZ89-R	5'-AGCAGAATTCTTATTTGCTTACGTGAATCTGACA-3'
pYZ90-R	5'-AGCAGAATTCTTATTTGCTTACGTGAATATCACA-3'
pYZ91-R	5'-AGCAGAATTCTTATTTGCTTACGTGAATACTAC-3'
Sumo-F	5'-GATATACCATGGGTCATCAC-3'
Sumo-R	5'-GCTAGGATCCATATCAGCAGCGGCGCCC-3'
pYZ81-F	5'-AGCAGGTACCATGAATAAAAAAAATATAAAAAGA-3'
pYZ81-R	5'-AGCTCTCGAGTCATTTCCTCTTCCCTCCTTTC-3'
pYX125-F	5'-CGGGGTACCATGATAAAAAGTTCATTTAAAGCTC-3'
pYX125-R	5'-CCGCTCGAGTCATTTCATGTATTCTTCCGAAAC-3'
pYX126-F	5'-CCGCTCGAGTGCTTAAGTCGAACAG-3'
pYX126-R	5'-CCGCTCGAGTCATTTCATGTATTC-3'
pYX105/106-F	5'-GTACCCTCGAGTGCTTAAGTCGAACAGAAAG-3'
pYX105/106-R	5'-CTCGACTCGAGTCATTTCCTCTTCCCTCCTTTC -3'
pYX122-F	5'-GATATGGATCCAATGAGTACAAAAGATTTTAAC-3'
pYX122-R	5'-AGCTCGAATTCTTAGTTGCCGAAGTGGCAAC-3'
pYX123-F	5'-GATATGGATCCAATGAGTACAAAAGATTTTAAC-3'
pYX123-R	5'-AGCTCGAATTCTTATTTCTTGCCAATACCCAC-3'

Primers of plasmids for nisin overexpression *in L. lactic*

pRL415-F	5'-GTAGCTTTTTAAATATGGGTCGATCTAATATCTTGATTTTCTAGTTCCTG-3'
pRL415-R	5'-TCCAAGTTAAAATCTTTTGTACTCATTTTGAGTGCCTCCTTATAATTTATT-3'
pRL415-VF	5'-AATAAATTATAAGGAGGCACTCAAAATGAGTACAAAAGATTTTAACTTGGA-3'
pRL415-VR	5'-CAGGAACTAGAAAATCAAGATATTAGATCGACCCATATTTAAAAAGCTAC-3'
pRL423-F	5'-GTAGTATTCACGTAAGCAAATAACCAAATCAAAGGATAGTATTTTGTTAG-3'
pRL423-R	5'-CTTGCATGCCTGCAGGTCGACTCTAGTCATTTCATGTATTCTTCCGAAAC-3'
pRL423-VF	5'-GTTTCGGAAGAATACATGAAATGACTAGAGTCGACCTGCAGGCATGCAAG-3'
pRL423-VR	5'-CTAACAAAATACTATCCTTTGATTTGGTTATTTGCTTACGTGAATACTAC-3'

Note: For plasmid construction via restriction enzyme digestion and ligation, complementary sequences were designed using the Primer Premier 5 software. Suitable restriction sites and protective bases were introduced. For plasmid construction via the Gibson cloning method, complementary sequences were designed using the Primer Premier 5 software and were flanked by the homologous sequence. The restriction sites used for cloning are underlined.

Table S5. Plasmids for Protein Purification

Plasmids	Replication origin	Overexpressed genes	Resistance	Reference
**Plasmids for CFPS biosynthesis**				
pJL1-*sfGFP*	pBR322	PT7: N-terminal His6-tagged *nisZ*	Kan	Zhang et al.[1]
pJL1-*nisZ*	pBR322	PT7: N-terminal His6-tagged *nisZ*	Kan	This study
pET28a-*nisB*	pBR322	PT7: N-terminal His6-tagged *nisB*	Kan	This study
pET28a-nisC	pBR322	PT7: N-terminal His6-tagged *nisC*	Kan	This study
pET28a-*nisP*	pBR322	PT7: N-terminal His6-tagged *nisP*	Kan	This study
pET28a-*nisPs*	pBR322	PT7: N-terminal His6-tagged *nisPs*	Kan	This study
pRL1	pBR322	PT7: N-terminal His6-tagged precursor peptide gene of RL1	Kan	This study
pRL2	pBR322	PT7: N-terminal His6-tagged precursor peptide gene of RL2	Kan	This study
pRL3	pBR322	PT7: N-terminal His6-tagged precursor peptide gene of RL3	Kan	This study
pRL4	pBR322	PT7: N-terminal His6-tagged precursor peptide gene of RL4	Kan	This study
pRL5	pBR322	PT7: N-terminal His6-tagged precursor peptide gene of RL5	Kan	This study
pRL6	pBR322	PT7: N-terminal His6-tagged precursor peptide gene of RL6	Kan	This study
pRL7	pBR322	PT7: N-terminal His6-tagged precursor peptide gene of RL7	Kan	This study
pRL8	pBR322	PT7: N-terminal His6-tagged precursor peptide gene of RL8	Kan	This study

pRL9	pBR322	PT7: N-terminal His6-tagged precursor peptide gene of RL9	Kan	This study
pRL10	pBR322	PT7: N-terminal His6-tagged precursor peptide gene of RL10	Kan	This study
pRL11	pBR322	PT7: N-terminal His6-tagged precursor peptide gene of RL11	Kan	This study
pRL12	pBR322	PT7: N-terminal His6-tagged precursor peptide gene of RL12	Kan	This study
pRL13	pBR322	PT7: N-terminal His6-tagged precursor peptide gene of RL13	Kan	This study
pRL14	pBR322	PT7: N-terminal His6-tagged precursor peptide gene of RL14	Kan	This study
pRL15	pBR322	PT7: N-terminal His6-tagged precursor peptide gene of RL15	Kan	This study
pRL16	pBR322	PT7: N-terminal His6-tagged precursor peptide gene of RL16	Kan	This study
pRL17	pBR322	PT7: N-terminal His6-tagged precursor peptide gene of RL17	Kan	This study
pRL18	pBR322	PT7: N-terminal His6-tagged precursor peptide gene of RL18	Kan	This study
pJL1-bagelicin	pBR322	PT7: N-terminal His6-tagged precursor peptide gene of bagelicin	Kan	This study
**Plasmids for mLanA overexpression in *E. coli***				
pYZ82	RSF	PT7: *nisB*	Kan	This study
pYZ85	RSF	PT7: *nisB* and N-terminal His6-tagged precursor peptide gene of RL6	Kan	This study

pYZ86	RSF	PT7: *nisB* and N-terminal His6-tagged precursor peptide gene of RL8	Kan	This study
pYZ87	RSF	PT7: *nisB* and N-terminal His6-tagged precursor peptide gene of S29A	Kan	This study
pYZ89	RSF	PT7: *nisB* and N-terminal His6-tagged precursor peptide gene of M5	Kan	This study
pYZ90	RSF	PT7: *nisB* and N-terminal His6-tagged precursor peptide gene of M4	Kan	This study
pYZ91	RSF	PT7: *nisB* and N-terminal His6-tagged NisZ	Kan	This study
pYZ92	RSF	PT7: *nisB* and N-terminal Sumo-tagged precursor peptide gene of M4	Kan	This study
pYZ93	RSF	PT7: *nisB* and N-terminal Sumo-tagged precursor peptide gene of M5	Kan	This study
pYZ95	RSF	PT7: *nisB* and N-terminal Sumo-tagged precursor peptide gene of RL6	Kan	This study
pYZ96	RSF	PT7: *nisB* and N-terminal Sumo-tagged precursor peptide gene of RL8	Kan	This study
pYZ97	RSF	PT7: *nisB* and N-terminal Sumo-tagged precursor peptide gene of S29A	Kan	This study

pYZ99	RSF	PT7: *nisB* and N-terminal Sumo-tagged NisZ	Kan	This study
pYZ81	P15A	PT7: *nisC*	CmR	This study
pYX106	RSF	PT7: *nisB* and N-terminal His-tagged precursor peptide gene of RL6, and PT7: *nisC*	Kan	This study
pYX125	P15A	PT7: *nisB*	CmR	This study
pYX126	P15A	PT7: *nisC* and PT7: *nisB*	CmR	This study
pYX105	RSF	PT7: *nisB* and N-terminal Sumo-tagged precursor peptide gene of S29A, and PT7: *nisC*	Kan	This study
pYX122	RSF	PT7: *nisB* and N-terminal Sumo-tagged precursor peptide gene of RL13, and PT7: *nisC*	Kan	This study
pYX123	RSF	PT7: *nisB* and N-terminal Sumo-tagged precursor peptide gene of RL14, and PT7: *nisC*	Kan	This study
**Plasmids for nisin overexpression in *L. lactis***				
pRL415	pWV01	Pnis: *nisZ*	Emr	This study
pRL423	pWV01	Pnis: *nisZ* and *nisB*	Emr	This study

References

[1] Y. Zhang, H. Qianyin, D. Zixin, X. Yancheng, L. Tiangang, *Biochem. Eng. J.* **2018**, 138, 47.
[2] G. Y. Tan, K. H. Deng, X. H. Liu, H. Tao, Y. Y. Chang, J. Chen, K. Chen, Z. Sheng, Z. X. Deng, T. G. Liu, *ACS Synth. Biol.* **2017**, 6, 995.
[3] B. Soufi, F. Gnad, P. R. Jensen, D. Petranovic, M. Mann, I. Mijakovic, B. Macek, *Proteomics* **2008**, 8, 3486.
[4] D. Field, M. Begley, P. M. O'Connor, K. M. Daly, F. Hugenholtz, P. D. Cotter, C. Hill, R. P. Ross, *PloS one* **2012**, 7; L. Zhou, A. J. van Heel, M. Montalban-Lopez, O. P. Kuipers, *Front. Cell Dev. Biol.* **2016**, 4; Q. Li, M. Montalban-Lopez, O. P. Kuipers, *Appl. Environ. Microb.* **2018**, 84.
